# Parameter estimation from amplitude collapse in correlated matter-wave interference

**DOI:** 10.1126/sciadv.aeg5334

**Published:** 2026-08-26

**Authors:** Daniel Derr, Dominik Pfeiffer, Ludwig Lind, Gerhard Birkl, Enno Giese

**Affiliations:** ^1^ Technische Universität Darmstadt, Fachbereich Physik, Institut für Angewandte Physik, Schloßgartenstraße 7, 64289 Darmstadt, Germany.; ^2^ Helmholtz Forschungsakademie Hessen für FAIR (HFHF), GSI Helmholtzzentrum für Schwerionenforschung, 64291 Darmstadt, Germany.

## Abstract

Operating matter-wave interferometers as quantum detectors for fundamental physics or inertial sensors with unprecedented accuracies relies on noise rejection, often implemented by correlating multiple sensors. They can be spatially separated (gradiometry or gravitational-wave detection) or consist of different internal states (magnetometry or quantum clock interferometry), with a signal-amplitude modulation serving as a signature of a differential phase. In this work, we introduce parameter estimation from amplitude collapse (PEAC) by applying statistical inference techniques for different magnetically sensitive substates of an atom interferometer. We demonstrate that PEAC provides higher trueness, resulting in a substantially reduced bias compared to standard methods for perfectly correlated signals, while achieving competitive precision near, but not at, vanishing amplitudes. This indicates that vanishing signals do not constitute the most favorable working point for high-accuracy sensing, relevant to quantum clock interferometry. PEAC presents a generally applicable complementary evaluation method for correlated interferometers without phase stability, increasing the overall accuracy and enabling applications beyond atom-based interferometry.

## INTRODUCTION

To exploit the full potential of atom interferometers as quantum sensors at the highest accuracy, the signal of interest is often encoded in the differential phase between two correlated interferometers. This strategy suppresses common-mode noise and is an established, yet crucial tool for high-precision measurements, e.g., setting new records in the determination of the fine-structure constant (*1*, *2*), forming the working principle of quantum-based gravitational-wave or dark-matter detectors (*3*–*5*) on large baselines (*5*, *6*) and being central to operational and proposed quantum tests of fundamental physics (*7*–*10*) and general relativity (*11*–*18*). It is also key for robust sensors (*19*, *20*) used in magnetometry (*21*–*24*), gyroscopy (*25*–*28*), or gradiometry (*29*–*31*) with applications in inertial sensing, exploration, and navigation (*27*, *32*). In such real-world applications, the differential phase can be extracted even in noisy environments, where the interference signal cannot be resolved in a single interferometer.

However, differential phase estimation in correlation measurements poses its own challenges even for established techniques such as fitting ellipses to parametric plots of noisy but correlated bivariate data that extract the phase from the ellipses’ eccentricity (*33*–*36*).

Because concepts for specifying measurement quality are not used consistently across subfields, we adopt the following definitions (*37*, *38*): Trueness denotes the closeness of the estimated (reconstructed) phase $\theta_{\text{rec}}$ to the true (e.g., set) phase $\theta_{\text{set}}$. As a practical quantitative measure of trueness, we consider the bias $\theta_{\text{bias}}=\theta_{\text{rec}}-\theta_{\text{set}}$. Precision characterizes the closeness of agreement of repeated estimates under identical conditions. We therefore choose the spread, i.e., the standard deviation associated with the estimation process, as a quantifiable measure. Accuracy refers qualitatively to the combined effect of trueness and precision. While typically precise, ellipse-based phase estimation becomes biased except for a circle (θ=π/2 and odd multiples). These are the only phases where the estimator is unbiased. For all other cases, a systematic error is introduced. The trueness of the phase estimate degrades strongly near degeneracy points (θ=πℤ), where the underlying ellipse collapses to a line.

In this work, we propose and implement parameter estimation from amplitude collapse (PEAC) as an additional, complementary statistical technique for noisy matter-wave interferometers and implement it for correlated interferometers in magnetometry and for velocimetry by deliberately inducing an amplitude collapse. We demonstrate, both experimentally and theoretically, that PEAC enhances the trueness by a bias reduction for a broad range of phase settings in magnetometry, albeit at the cost of precision, yielding overall improved accuracy also near the degeneracy points, i.e., for perfectly in-phase or π-phase–shifted correlated interferometers.

PEAC is inspired by quantum clock interferometry (*39*–*44*), where a superposition of internal states undergoes an atom-interferometer sequence, creating a spatial superposition in which possible relativistic (*45*, *46*) and proper-time effects (*47*) encode a periodic signal-amplitude loss associated with which-path information (*48*). Here, the internal states acquire different interferometer phases, e.g., due to relativistic effects associated with mass-energy equivalence, but are not measured individually. In particular, atoms in different internal states have slightly different rest masses due to their internal energy, known as mass defect (*49*, *50*), which leads to state-dependent phase evolution in the interferometer. Although these differential phase shifts are typically minute and can, in certain situations, also be extracted using the methods discussed below, the question remains how such a signature can be most effectively used in quantum clock interferometry. Recent experiments (*51*) operate beyond the perturbative regime, with differential phases induced by magnetic field gradients, a mechanism that we also use in the present work.

Similar to those quantum-clock schemes, PEAC does not necessarily require measuring correlated interferometers individually but relies on the detection of (possibly incoherent) mixtures of them. A differential phase shift between the individual interferometers induces a beating of their superposed interference fringes. This characteristic structure manifests as collapses and revivals of the combined signal’s oscillation amplitude, whose pattern appears in statistical histograms of population measurements, extractable (*32*, *52*–*54*) even when suffering large phase noise.

We analyze the PEAC performance in atom interferometers containing a stochastic mixture of magnetically sensitive substates to estimate the acceleration induced by a magnetic-field gradient. Using state-selective measurements, we compare this technique to corresponding ellipse fits, demonstrating that PEAC provides a complementary statistical analysis of existing correlation data not through joint phase fitting but via an optimally chosen transformation of the bivariate coordinates and their amplitude marginal distributions. Such an analysis can be always performed and optimized in addition to ellipse fits. PEAC demonstrates improved overall performance, with the highest accuracy for phases near, but not exactly at degeneracy points, in contrast to expectations from Gaussian uncertainty propagation. The signal amplitude is small but still finite, providing a working point that exhibits negligible bias and precision comparable to ellipse fitting. This observation highlights a distinct operating regime that differs from geometric phase amplification (*51*).

The method extracts a measurand, in our case the differential phase of interest, from histograms of a suitably chosen linear combination of correlated signals. It therefore requires a model for the dominant noise and for the functional dependence of the probability distribution on the measurand, in our case through the amplitude of an interference pattern. The method also depends on the weights or mixture fractions that define the combined signal. These weights, or other distribution-specific parameters, may be determined either by scanning the measurand or, if this is experimentally inaccessible, from independent experiments, as demonstrated below.

PEAC applies to a wide range of cases: coherent implementations such as quantum clocks and incoherent, stochastic mixtures of components. It allows extracting phase information in setups previously considered unsuitable, e.g., when these components spatially overlap. At the same time, it can also be applied in situations where components can be individually resolved, a necessary condition for ellipse fitting. Moreover, PEAC enables phase extraction even when phase fluctuations prevent fringe resolution in correlated interferometer signals (*55*, *56*) and when the differential phase cannot be tuned, providing a valuable toolbox for next-generation platforms (*13*, *20*, *21*, *52*, *54*, *57*) for matter-wave sensors with ultracold quantum gases. Furthermore, we extend the approach to related estimation problems in which information is encoded in contrast or envelope parameters, such as the extraction of effective temperatures or rotation rates from contrast-envelope fitting in atom interferometry (*58*–*60*). Last, it can be combined with any statistical inference technique, such as least-squares fitting or maximum likelihood estimation.

## RESULTS

### PEAC methodology

PEAC is an estimation method applicable to interferometric experiments that extracts the value of a parameter of interest from the amplitude of an interference signal without the need to resolve an individual interference fringe. We demonstrate this for two distinct parameters: the differential phase between correlated interferometers, extracted from the characteristic amplitude collapse of a combined signal for magnetometry in the “Magnetometry” section, and the momentum width of an atomic cloud, extracted from the decreasing amplitude upon deliberately opening the interferometer to reduce the clouds’ overlap in the “Velocimetry” section.

The parameter of interest is extracted from a statistical model with a known probability density function (PDF), which is experimentally probed by repeated measurements used to construct histograms. The PDF can be fitted directly to these histograms or one can use more sophisticated procedures such as maximum likelihood estimation to extract the PDF’s parameters (see the Supplementary Materials).

The PDF generally depends not only on the parameter of interest but also on additional experimental quantities, such as the intrinsic contrast. If a control parameter is experimentally accessible that induces a characteristic variation of the amplitude, then PEAC can be operated in scanning mode, permitting the simultaneous extraction of both intrinsic parameters and the quantity of interest. We demonstrate this mode of operation by scanning the differential phase for magnetometry and the timing asymmetry for velocimetry, from which the respective quantities of interest are extracted via a fit to the amplitude model.

If no such control parameter is accessible, but the intrinsic parameters of the PDF are sufficiently characterized by independent measurements, then PEAC is operated in static mode, allowing the quantity of interest to be estimated from a single experimental configuration. We demonstrate static operation in the “Correlation measurements—Connection to ellipse fitting” section, where we independently determine the intrinsic contrast from the individual signals of two correlated interferometers, which is then used to infer the magnetic field gradient without the necessity to scan the differential phase.

#### Magnetometry

**Fig. 1.**
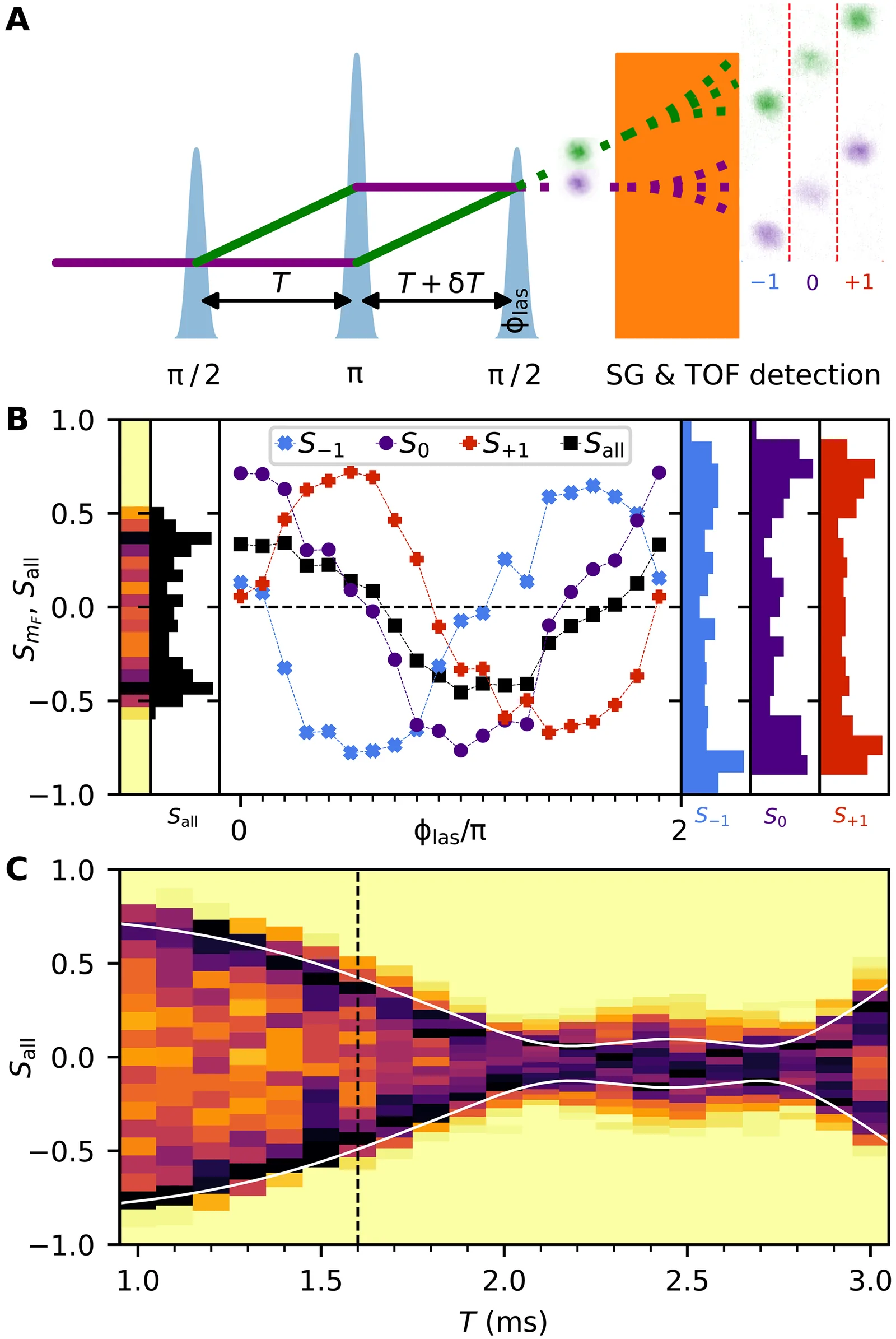
Spacetime diagram and signal extraction. (**A**) Mach–Zehnder atom interferometer (MZI) realized by three Bragg pulses separated by T and T+δT, respectively, driving transitions between momentum states $|0\hbar k_{\text{eff}}\rangle$ (purple) and $|1\hbar k_{\text{eff}}\rangle$ (green). The phase of the final pulse is shifted by $\phi_{\text{las}}$, thus scanning the interference fringes at the two exit ports. The MZI is followed by a TOF that spatially separates the exits, during which an optional Stern–Gerlach (SG) field can separate the $m_{F}$ substates of the F=1 ground state within each momentum class in addition. (**B**) Fringe scans (20 settings of $\phi_{\text{las}}$, 15 repetitions averaged, uncertainties omitted for visual clarity) at T=1.6 ms. Without the SG field, we observe the fringe $S_{\text{all}}$ (black), with the corresponding histogram from 300 measurements (left marginal, also displayed as a density plot on the far left) has a double-peak structure that depends on the signal’s amplitude. Applying an SG field allows resolving the individual signals $S_{m_{F}}$ per substate (blue, indigo, and red), whose histogram distributions are shown at the right marginal. The individual signals show equal amplitudes, while the amplitude of $S_{\text{all}}$ is reduced due to summation of the individual $S_{m_{F}}$ with shifted phases. (**C**) Combined density plot of the histograms of $S_{\text{all}}$, obtained for a scan of the interferometer time T, reveals the collapse and revival of the amplitude caused by the incoherent overlay of the three signals $S_{m_{F}}$. The white lines trace the calculated amplitude as $\pm A_{\text{all}}$, parametrized by Eq. 3. The black dashed line corresponds to the data shown in (B).

In this subsection, we illustrate PEAC in scanning mode for a concrete experimental implementation exploiting the connection between the beat amplitude and a differential phase in a three-level system, as well as the associated level populations. Specifically, we apply PEAC to atom interferometry-based magnetometry (*21*–*24*) in one dimension, a benchmark application well-suited to typical vibration-prone environments and compact devices, even when state-selective interferometry is unavailable. Ultracold ^87^Rb atoms from a Bose–Einstein condensate (BEC) provide a suitable point source (*32*) with subrecoil momentum width, coherence, and access to efficient Bragg diffraction (*61*). Our experiment (*62*) creates BECs in a far-detuned crossed optical dipole trap (CDT), typically with 20,000 atoms at $20\left(5\right)\;\mathrm{nK}$ and a condensate fraction exceeding 80%. When no optical pumping or magnetic quantization is applied, the atoms occupy a stochastic mixture of all magnetic substates $m_{F}\in \left\{-1,0,+1\right\}$ of the ground-state manifold $|5^{2}S_{1/2},F=1\rangle$. All states are addressed and Bragg-diffracted by a pair of counter-propagating laser beams (*63*) with a frequency difference Δω=2π×15.084 kHz, coupling the two momentum states $|0\hbar k_{\text{eff}}\rangle$ and $|1\hbar k_{\text{eff}}\rangle$ with the modulus $k_{\text{eff}}=2\times 2\pi /780.226\;\text{nm}$ of the effective wave vector of the two-photon Bragg transition. With these Bragg pulses, we implement a Mach–Zehnder atom interferometer (MZI) in a π/2−π−π/2 pulse configuration as illustrated in Fig. 1A. The laser-intensity envelopes of all three pulses are shaped as Blackman window pulses (*64*, *65*) of length τ=100 μs. We label the temporal separation of the maxima of two consecutive pulses by T and T+δT, respectively. Identical values T∈[1 ms,3 ms] and δT=0 result in a total duration of the MZI sequence up to 2T+τ=6.1 ms.

After the symmetric MZI, a time of flight (TOF) spatially separates the interferometer output ports associated with the momentum states $|j\hbar k_{\text{eff}}\rangle$ for measuring the atom numbers $N_{j}\left(\phi \right)=B_{j}\pm A_{j}\text{cos}\phi$ in different momentum classes j∈{0,1}, with the interferometric phase ϕ, baseline $B_{j}$, and amplitude $A_{j}$. We define the interferometer signal as the normalized population difference, i.e.,

$$S_{\text{all}}\left(\phi \right)=\frac{N_{0}\left(\phi \right)-N_{1}\left(\phi \right)}{N_{0}\left(\phi \right)+N_{1}\left(\phi \right)}≅B_{\text{all}}+A_{\text{all}}\text{cos}\phi$$ (1)

where the signal’s baseline $B_{\text{all}}$ and amplitude $A_{\text{all}}$ satisfy $|B_{\text{all}}|+|A_{\text{all}}|\leq 1$ and depend on $B_{j},A_{j}$. For more details on the experimental apparatus and the derivation of $S_{\text{all}}$, we refer to Materials and Methods.

By characterizing $S_{\text{all}}$, for example, by scanning an interferometer fringe, one can extract the interferometric phase ϕ and thus the quantity of interest influencing ϕ. For instance, this could be the projection $a_{\text{ext}}$ of an external acceleration in the direction of the Bragg beams. To scan an interference fringe, we offset the phase of the final π/2 pulse by 20 evenly spaced values of $\phi_{\text{las}}\in \left[0,2\pi \right)$. We perform 15 repetitions per T for improved signal to noise. A typical fringe for T=1.6 ms is shown in the center panel of Fig. 1B by the black trace. We see a clear oscillation and periodicity of the fringe characterized by its amplitude $A_{\text{all}}$, phase ϕ, and baseline $B_{\text{all}}$. If ϕ remains stable between all pulses, then a cosine can be fitted to the fringe, providing direct access to these parameters. Conversely, in the absence of phase stability, the amplitude $A_{\text{all}}$ and baseline $B_{\text{all}}$ of $S_{\text{all}}$ can be obtained via a statistical analysis (*52*, *54*, *66*, *67*), albeit at the cost of losing the phase information. This procedure also can be applied to data obtained under phase-stable conditions, such as our measurements at small T.

To demonstrate this statistical method, we show, at the left margin of Fig. 1B, a histogram associated with the fringe in black, where the statistics is generated from all 300 contributing measurements. The very left column shows the same histogram converted into a density plot using false-color coding. We observe the typical double-peak structure of the histogram (*52*, *66*), where the distance of the peaks is roughly given by $2A_{\text{all}}$ and their individual width is determined by baseline fluctuations of $B_{\text{all}}$. This characterization is valid for a homoscedastic signal, where $A_{\text{all}}$ does not fluctuate, as specified in Materials and Methods, which is dominated by technical noise and operates well above the quantum-projection noise limit.

In addition to scanning ϕ via $\phi_{\text{las}}$ for a fixed value of T, we also vary the interferometer time T∈[1 ms,3 ms] in steps of 100 μs. The corresponding histograms are displayed as a composite density plot in Fig. 1C, where each column is obtained in the same way as in Fig. 1B corresponding to the histogram behind the dashed black line at T=1.6 ms. We observe a decrease of the signal amplitude for increasing T with a collapse at T=2.1 ms, which is not due to loss of coherence, since $A_{\text{all}}$ revives for longer times. This behavior is caused by an underlying modulation of $A_{\text{all}}\left(T\right)$, which is a consequence of the beat of the three signals $S_{m_{F}}\left(\phi_{m_{F}}\right)$ defined in analogy to Eq. 1 and associated with three $m_{F}$ substates. The $m_{F}$ substates experience a state-dependent acceleration, partly due to a magnetic field gradient, acquiring different phases $\phi_{m_{F}}$, which induce this characteristic collapse and revival behavior. The magnetically insensitive $m_{F}=0$ substate acquires the phase $\phi_{0}$, including $\phi_{\text{las}}$ and accelerations and perturbations common to all substates. The magnetically sensitive states with $m_{F}\in \left\{+1,-1\right\}$ pick up the phases $\phi_{\pm 1}=\phi_{0}\pm \theta /2$ with the differential phase

$$\theta ≅2\;k_{\text{eff}}a_{\text{ext}}T^{2}\left(1+0.1486\;\tau /T\right)$$ (2)

between the two substates, induced by a Zeeman force that accelerates the $m_{F}=+1$ state by $a_{\text{ext}}$ in the direction of the Bragg beams and the $m_{F}=-1$ state by the same magnitude but in the opposite direction. Here, we correct the conventional MZI phase for finite-pulse durations (*68*) in τ/T, which can approach 0.1 in our case with τ=100 μs for short interferometer times. We present the derivation of the numerical factor in Materials and Methods.

In our experiment, we have additional access to the individual substates by applying the Stern–Gerlach (SG) method (*69*, *70*) during a TOF after the MZI: In each momentum exit port, we can create a spatial separation of the substates by applying an additional magnetic field gradient as depicted in Fig. 1A (right). This method allows us to determine the oscillatory signal $S_{m_{F}}$ of the individual substates as displayed in the center panel of Fig. 1B, along with the corresponding histograms at the right marginals. As expected, we observe the same oscillation amplitude $A_{m_{F}}$ for all three substates, which is in particular larger than $A_{\text{all}}$. Based on this insight, we conclude that in a nonstate-selective setting, the observable is $S_{\text{all}}=\lambda_{+1}S_{+1}+\lambda_{0}S_{0}+\lambda_{-1}S_{-1}$, where $\lambda_{m_{F}}$ are the weights of each substate in the incoherent stochastic mixture.

In the remainder of this section, we focus on this nonstate-selective setting. Here, the observable $S_{\text{all}}$ can be written as $S_{\text{all}}=B_{\text{all}}+A_{\text{all}}\text{cos}\left(\phi_{0}+\theta_{\text{off}}\right)$. The offset $\theta_{\text{off}}$ can be interpreted as a geometric phase (*51*) and depends on θ and $\lambda_{m_{F}}$ (Materials and Methods). Here, $B_{\text{all}}=\sum_{m_{F}}\lambda_{m_{F}}B_{m_{F}}$ is the baseline, and

$$A_{\text{all}}\left(\theta \right)=A_{0}\sqrt{\Delta \lambda^{2}{\text{sin}}^{2}\left(\theta /2\right)+{\left[\lambda_{0}+\left(\lambda_{+1}+\lambda_{-1}\right)\text{cos}\left(\theta /2\right)\right]}^{2}}$$ (3)

is the amplitude, with the population imbalance $\Delta \lambda =\lambda_{+1}-\lambda_{-1}$, assuming $A_{-1}=A_{0}=A_{+1}$. In this particular setting, we cannot access the differential phase θ directly from a histogram recorded for a single interferometer time T, since the weights $\lambda_{m_{F}}$ are unknown. However, scanning the interferometric time T allows to infer θ. The underlying reason is the characteristic variation of the amplitude $A_{\text{all}}$ with T, as evident from Eqs. 2 and 3, due to the overlay of the three individual interferometer signals with their respective phases. Crucially, the technique does not intrinsically require a scan of θ, provided the auxiliary parameters $A_{0}$ and $\lambda_{m_{F}}$ are constrained by independent measurements or deterministic tuning, as is done in quantum clock experiments. In this case, the static mode of PEAC can be used to reconstruct the phase for each experimental setting.

In the present section, PEAC in its scanning mode is explicitly performed by fitting the histogram’s PDF for each T to obtain $A_{\text{all}}\left(T\right)$, where the form of the PDF (*52*, *54*, *67*) and further details can be found in Materials and Methods. Using $A_{\text{all}}\left(T\right)$, we fit Eq. 3 with the help of Eq. 2 to extract the acceleration and further parameters $a_{\text{ext}}=32.2(1)\;\text{mm}/s^{2}$, $\lambda_{0}=0.42\left(1\right)$, Δλ=0.18(1), and $A_{0}=0.79\left(1\right)$. We calculate the modulated amplitude $A_{\text{all}}\left(T\right)$ from these values and plot $\pm A_{\text{all}}\left(T\right)$ as white lines in Fig. 1C. The uncertainty of $a_{\text{ext}}$ and all other experimental uncertainties in the following are obtained via bootstrapping (*71*–*73*), as explained in Materials and Methods.

Even in the presence of phase instability and a nonstate-selective setup, PEAC allows one to estimate the differential phase θ and thus $a_{\text{ext}}$. We emphasize that phase information cannot be extracted from a single histogram when the intrinsic PDF parameters are not independently known; however, by considering multiple times T, the collapse of the amplitude $A_{\text{all}}$ enables the extraction of the phase. For comparison, as we have direct access to $\lambda_{m_{F}}$ from SG population measurements, we directly obtain $\lambda_{0}=0.39\left(6\right)$, $\lambda_{-1}=0.21\left(3\right)$, $\lambda_{+1}=0.40\left(4\right)$, Δλ=0.19(5), and $A_{0}=0.83\left(1\right)$, averaged over all T values. These measured populations vary with every experimental realization and are unknown, if no state-selective detection is performed. While there are drifts on longer timescales, the population mixture appears constant within experimental uncertainties on the timescale of the performed experiments. Here, $A_{0}$ is the average amplitude of the $m_{F}$ substates obtained by fitting the individual $m_{F}$ histograms. These results are in good agreement with those obtained using PEAC.

If the populations $\lambda_{m_{F}}$ and amplitude $A_{0}$ are known, e.g., from other, independent calibration measurements or the state preparation procedure, then the differential phase can be estimated from a single set of data for a fixed interferometer time T, so that no control over the differential phase is necessary to implement PEAC. With these quantities at hand, Eq. 3 can be applied pointwise, so that we demonstrate PEAC in static mode in the “Correlation measurements—Connection to ellipse fitting” section. Last, we highlight that PEAC can even be applied to systems with more than three states, albeit at the cost of a richer beat structure of the amplitude $A_{\text{all}}$ (Materials and Methods).

#### Velocimetry

**Fig. 2.**
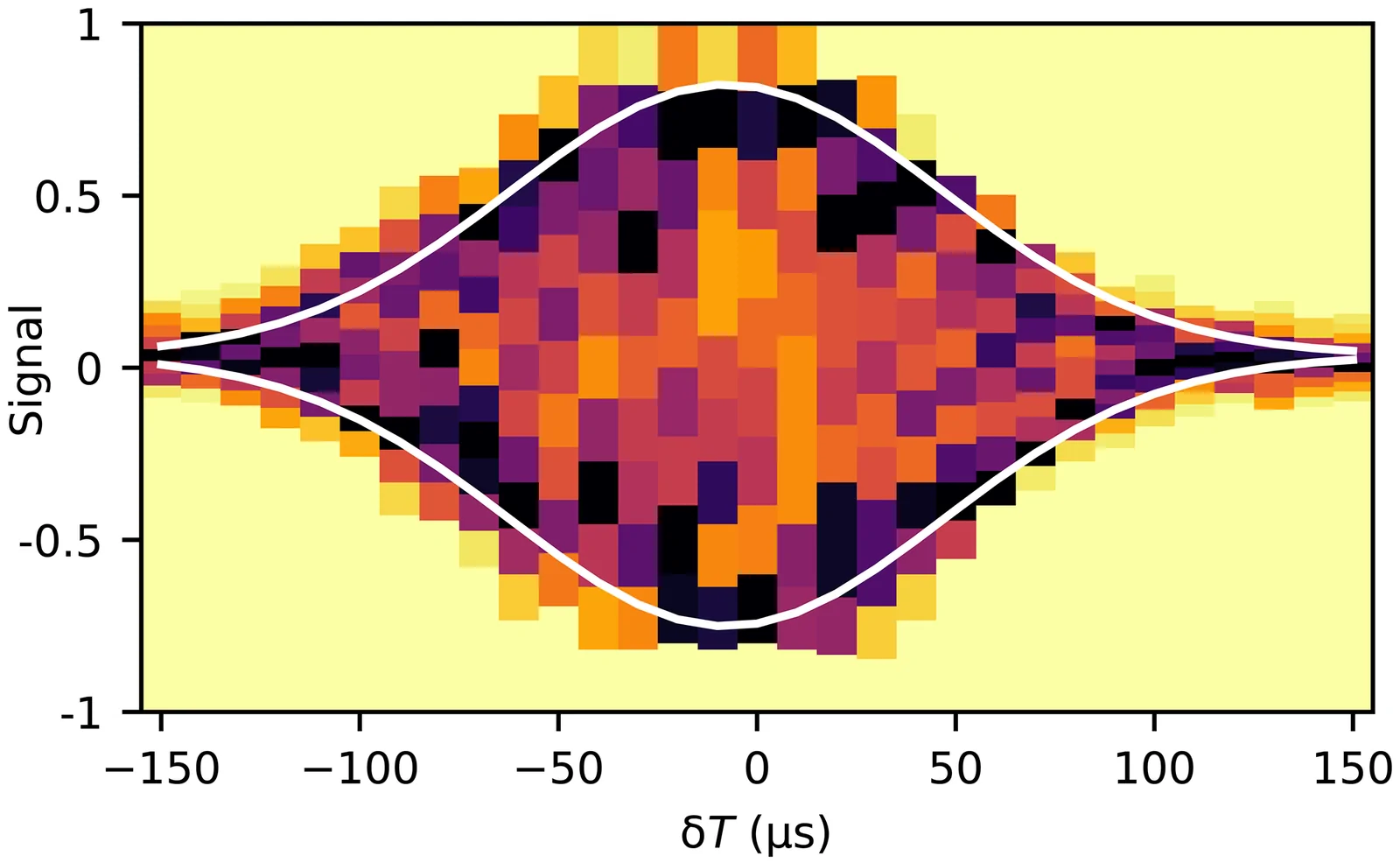
Amplitude collapse in an open interferometer. Density plot of an open interferometer signal for the $m_{F}=0$ component. Increasing timing asymmetry ∣δT∣ reduces the overlap of the two interferometer arms in the output, leading to an amplitude collapse. The white solid line traces the envelope of the amplitude collapse used to extract the momentum width of the atomic cloud.

In the previous section, the observed amplitude collapse was caused by a beat signal of three correlated interferometer signals encoding a differential phase. We now demonstrate the broad applicability of PEAC by performing velocimetry of atomic clouds to determine their momentum width Δp. Since we expect the same behavior for all $m_{F}$ substates, we restrict the analysis to the $m_{F}=0$ component extracted from a state-selective detection via the SG method. Similar to contrast envelope fitting (*58*–*60*), the interferometer is deliberately opened (*74*) according to Fig. 1A by shifting the last π/2 pulse by δT∈[−150 μs,150 μs] with a step size of 10 μs. We operate the interferometer at a fixed value of T=3 ms and record five phase scans as described in the “Magnetometry” section, totalling to 100 realizations per δT. The timing asymmetry reduces the spatial overlap of the wave packets propagating along the two arms at the output ports. The resulting displacement scales as $\delta \zeta =\hbar k_{\text{eff}}\delta T/m$ with the mass m of ^87^Rb atoms. Assuming a Gaussian momentum distribution with width Δp, the amplitude of the interference signal $A\left(\delta T\right)=A_{0}\text{exp}\left[-{\delta \zeta }^{2}\Delta p^{2}/\left(2\hbar^{2}\right)\right]=A_{0}\text{exp}\left[-\delta T^{2}/\left(2T_{c}^{2}\right)\right]$ is suppressed by a Gaussian envelope with an apparent coherence time $T_{c}=m/\left(k_{\text{eff}}\Delta p\right)$. Similar to magnetometry, we apply PEAC by extracting the amplitude A(δT) from fits of the PDF to the histograms of the interferometer signal, again employing thousandfold bootstrapping. These amplitudes are then fitted to the Gaussian envelope to extract $T_{c}$. In Fig. 2, we show the density plots of the resulting histograms with white solid lines tracing the envelope A(δT) for the averaged fit parameters. We obtain $A_{0}=0.79\left(2\right)$ and $T_{c}=54.4\left(15\right)\;\text{μs}$, with the center of the Gaussian shifted by 7.6(1.5) μs. Given this apparent coherence time, we estimate the rms momentum spread as $\Delta p=0.100(3)\;\hbar k_{\text{eff}}$, which translates to an effective temperature of $\Delta p^{2}/\left({\mathrm{mk}}_{B}\right)=13.7(7)\;\text{nK}$. These results agree with the widths obtained by TOF measurements ($\Delta p=0.12(1)\;\hbar k_{\text{eff}}$), with the remaining difference attributed to a broadening of the ensemble by resonant absorption imaging and contributions of the thermal component (*75*). Using the timing asymmetry δT as a control parameter, PEAC therefore enables the determination of the momentum width of an atomic ensemble from the collapse of the amplitude in a deliberately opened interferometer.

### Correlation measurements—Connection to ellipse fitting

As demonstrated in the “Magnetometry” section, PEAC can be used to extract differential phases from the overlay and resulting beat of correlated signals in the scanning mode. Here, we turn to the static mode establishing a connection to ellipse fitting. In addition to emphasizing the application to systems without state-selective measurement capabilities, we now shift our focus to correlated systems in a setup that allows for state selection, where all intrinsic parameters of the PDF have been obtained from independent measurements. In this case, no control over the quantity of interest, i.e., the differential phase θ, is required, and PEAC can be applied to each dataset at fixed interrogation time T. Acknowledging that correlation measurements also can be carried out in systems with more than two distinct components (*41*, *76*, *77*), we restrict our evaluation to a two-state system, similar to typical quantum clocks using π/2 pulses to generate coherent superpositions between two internal states. We immediately can investigate this in our system by applying the SG method to distinguish the signals $S_{m_{F}}$ and choosing a symmetric sum of the $m_{F}=\pm 1$ states while excluding the magnetically insensitive $m_{F}=0$ state ($\lambda_{0}=0$). The summed signal

$$S_{\text{sum}}=\left(S_{-1}+S_{+1}\right)/\sqrt{2}$$ (4)

is scaled by choosing $\lambda_{\pm 1}=1/\sqrt{2}$, anticipating Eq. 6. Evaluating Eq. 3 for this system and δT=0 gives the amplitude modulation (*45*, *47*)

$$A_{\text{sum}}=\sqrt{2}A_{0}\left|\text{cos}\left(\theta /2\right)\right|$$ (5)

**Fig. 3.**
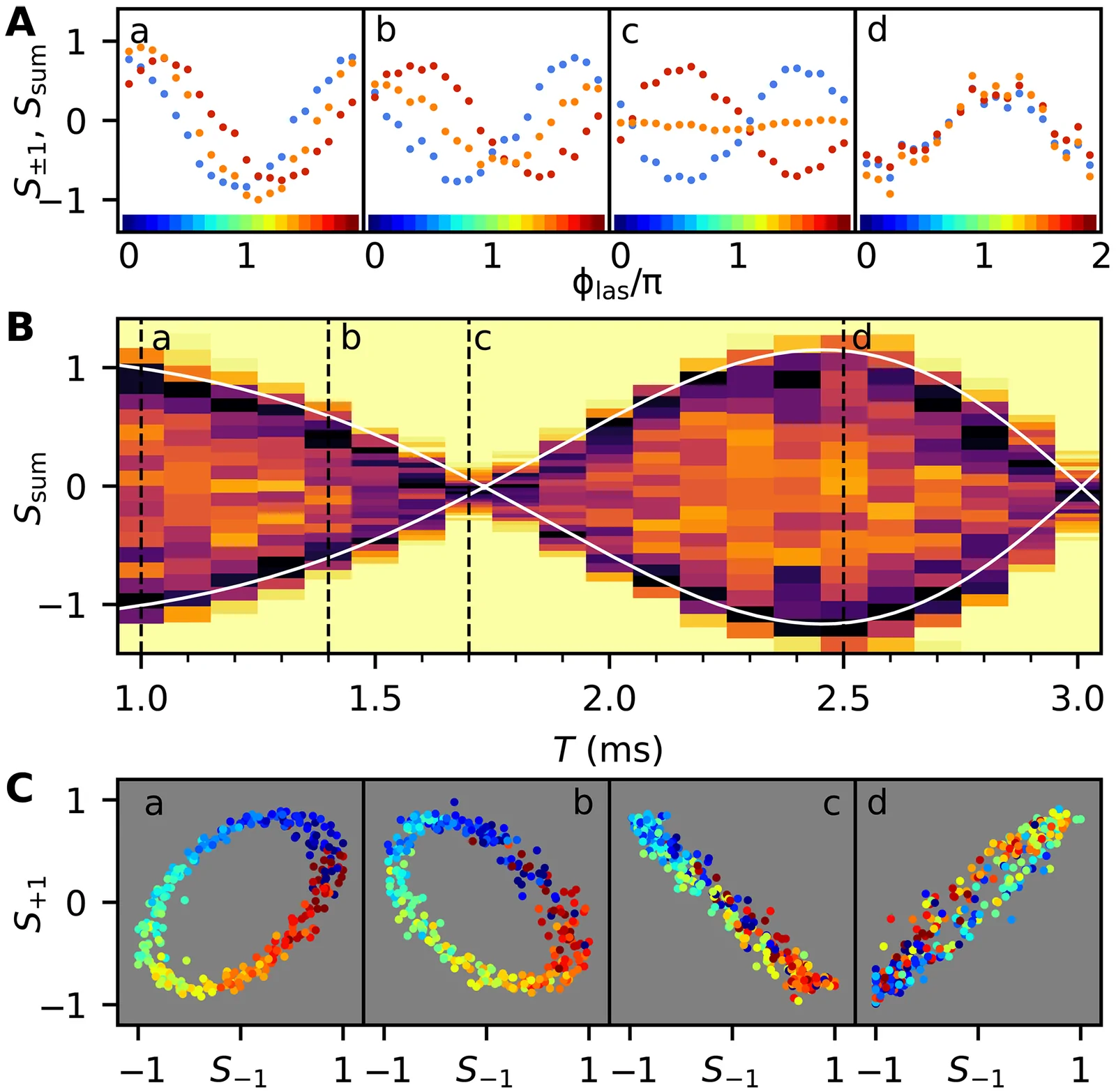
Interference fringes, amplitude collapse, and signal correlation. (**A**) Colored dots in panels (a) to (d) show the averaged interference fringes of $S_{+1}$ (red), $S_{-1}$ (blue), and $S_{\text{sum}}$ (orange) for four different values of T, i.e., for distinct differential phases θ. The beat of $S_{+1}$ and $S_{-1}$ leads to a collapsing amplitude of $S_{\text{sum}}$ for θ≅π (c). Uncertainties are omitted for visual clarity. (**B**) Collapse and revival of the amplitude $A_{\text{sum}}$ (white) as a function of T, inferred from the underlying histograms of $S_{\text{sum}}$ shown as density plot, where darker regions correspond to higher bin counts. The collapses occur at the beat nodes around T=1.7 ms and T=3 ms. Dashed black lines indicate the times (a) to (d) from (A). (**C**) Bivariate scatter plots for times (a) to (d) of the correlated signals $S_{+1}$ (vertical) and $S_{-1}$ (horizontal), with a color coding as in (A) indicating the phase scan of $\phi_{\text{las}}$. The eccentricity of the emerging ellipse encodes the differential phase θ. At T=1.7 ms (θ≅π) and T=2.5 ms (θ≅2π), the ellipses become degenerate and collapse to lines along the antidiagonal and diagonal, respectively.

In panels (a) to (d) of Fig. 3A, we plot the averaged fringes of the individual signals $S_{\pm 1}$ and $S_{\text{sum}}$ for four distinct interferometer times T∈{1.0 ms, 1.4 ms, 1.7 ms, 2.5 ms}, i.e., different phases θ. For increasing T, phase noise induces a reduction in the observed fringe amplitudes of $S_{+1}$ and $S_{-1}$, which are equal in magnitude. The observed fringe amplitude of $S_{\text{sum}}$ decreases accordingly but is in addition modulated by ∣cos(θ/2)∣ and depends on the phase difference.

The signals $S_{\pm 1}$ range from being slightly (a) and notably (b) out of phase via a vanishing $S_{\text{sum}}$ in (c) for a differential phase θ≅π to a rephasing at θ≅2π in (d). Resorting again to PEAC, we show the corresponding density plot of the histograms in Fig. 3B, with black dashed lines marking the chosen times from Fig. 3A. This visualization shows the collapse and revival of the amplitude $A_{\text{sum}}$ with T and by that with varying differential phase θ. The beat node of the signal at T=1.7 ms (θ≅π) becomes visible, with a secondary node discernible at T=3 ms (θ≅3π). Fitting the histogram PDF to $S_{\text{sum}}$ for each interferometer time T returns the time-dependent amplitude $A_{\text{sum}}\left(T\right)$, while fits to $S_{\pm 1}$ can be used to obtain $A_{0}$. The white line in Fig. 3B shows the envelope given by Eq. 5 for averaged values of $A_{0}=0.82\left(1\right)$ and $a_{\text{ext}}=32.2(1)\;\text{mm}/s^{2}$, obtained by bootstrapping the data and fitting. This envelope corresponds to the intrinsic amplitude of the signal, which is larger than the observed fringe amplitude, as the analysis of histograms is robust against phase noise.

Beside fitting the amplitude modulation, a known $A_{0}$ allows the pointwise estimation of the differential phase θ(T) by inverting Eq. 5. To account for the ambiguity of the inverted cosine function, we unwrap the phase. Fitting Eq. 2 to the reconstructed phases returns an external acceleration of $a_{\text{ext}}=32.2(1)\;\text{mm}/s^{2}$, which is in excellent agreement with our prior results.

We compare PEAC to a more established evaluation scheme for correlated systems based on parametric plots and fitting ellipses (*33*–*35*, *78*–*81*). The correlated signals $S_{\pm 1}$ for the interferometer times of Fig. 3A are visualized in a bivariate scatter plot in Fig. 3C, with $S_{-1}$ along the horizontal and $S_{+1}$ along the vertical axis; each measurement realization is shown as a colored dot. The color is chosen to represent the adjusted phase $\phi_{\text{las}}$, which results in a color gradient for the performed phase scan as can be seen in subfigures (a) to (d). For larger interferometer times T>1.6 ms, the color gradient becomes disordered, showing a transition to the phase-unstable regime of the experiment. While a scan of the phase $\phi_{\text{las}}$ traces the ellipse, the differential phase is encoded in its eccentricity, which we extract from geometric ellipse fitting routines (*21*, *82*–*84*). A detailed explanation of our fitting routine can be found in Materials and Methods. With this approach, we estimate an external acceleration of $a_{\text{ext}}=32.12(2)\;\text{mm}/s^{2}$, confirming the results obtained by PEAC.

In fact, both methods are inherently connected: The principal axes of the ellipses in Fig. 3C [(c) and (d)] are given by the diagonal and antidiagonal (see Materials and Methods for more details). The diagonal is proportional to the sum of the bivariate coordinates $\left(S_{+1}+S_{-1}\right)$, while the antidiagonal is proportional to their difference $\left(S_{+1}-S_{-1}\right)$. Rotating the bivariate coordinate system by the angle α leads to the definition of the general signal

$$S\left(\alpha \right)=S_{-1}\text{cos}\alpha +S_{+1}\text{sin}\alpha$$ (6)

**Fig. 4.**
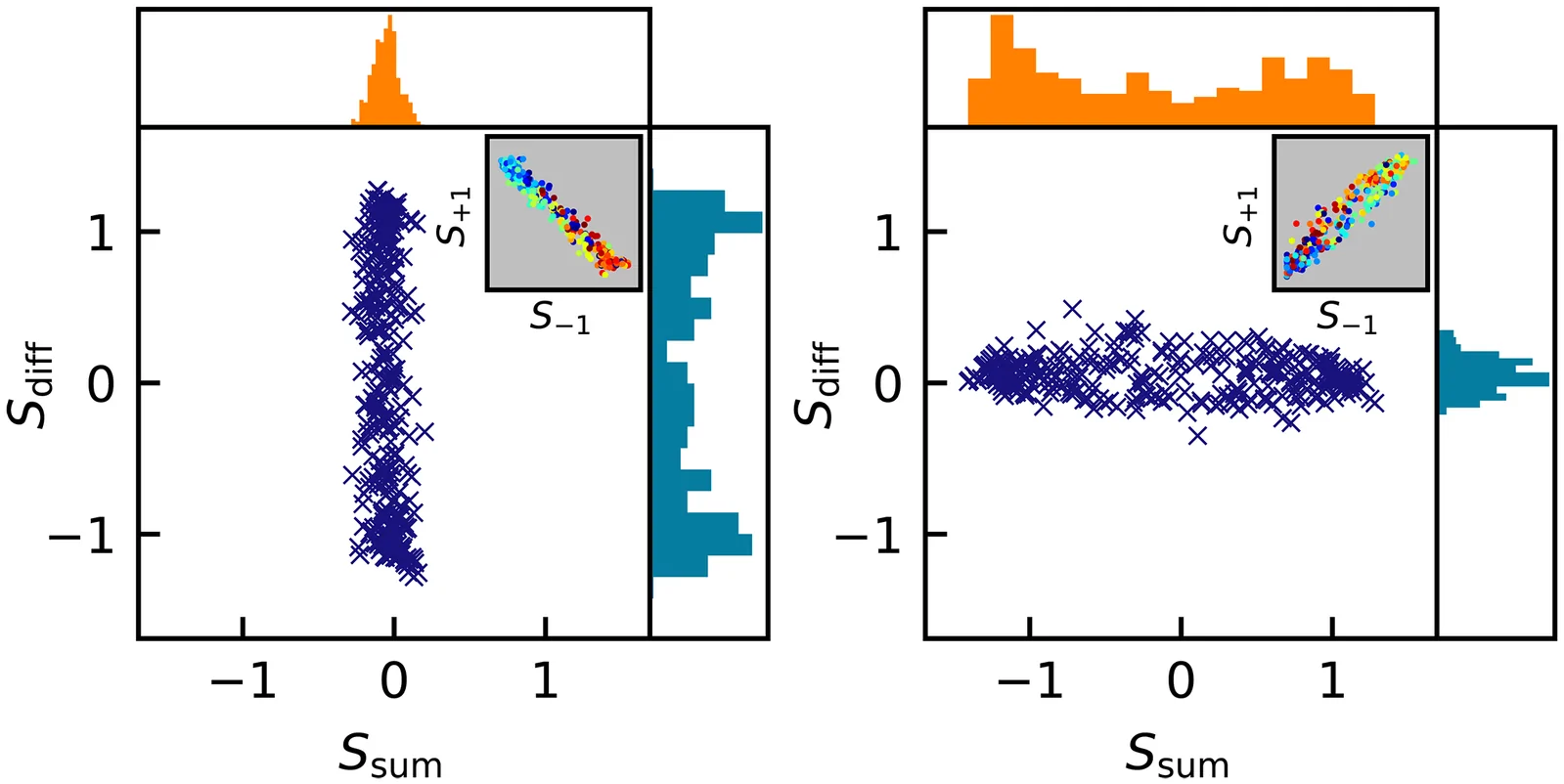
Rotated bivariate scatter plots and histograms. Rotating the original correlation signals (insets) for T=1.7 ms (θ≅π, left) and T=2.5 ms (θ≅2π, right) by π/4 clockwise aligns the ellipses to their principal axes, which correspond to $S_{\text{sum}}$ (horizontal) and $S_{\text{diff}}$ (vertical). The marginal distributions show the histograms of $S_{\text{sum}}$ and $S_{\text{diff}}$, illustrating that the two exchange their roles at the respective degeneracy points of θ. This representation visualizes the intrinsic link between ellipse-based estimation based on bivariate fitting and PEAC applied to $S_{\text{sum}}$ and $S_{\text{diff}}$. Note that $S_{\text{diff}}$ is only accessible by state-selective detection.

Hence, the special case $S_{\text{sum}}=S\left(\pi /4\right)$ denotes the axis along the diagonal and can be accessed even without state-selective measurements for two-component systems. A second case is $S_{\text{diff}}=S\left(3\pi /4\right)$, oriented along the antidiagonal, which can only be inferred in a state-selective setup. Consequently, for two correlated states such as presented here, applying PEAC generalizes to performing a statistical histogram analysis of the bivariate data along any angle α. In Fig. 4, we depict the ellipses aligned to their principal axes $S_{\text{sum}}$ and $S_{\text{diff}}$, i.e., rotated by π/4 clockwise, for T=1.7 ms (left) and T=2.5 ms (right) in the bivariate variables $\left(S_{\text{sum}},S_{\text{diff}}\right)$ with complementing histograms at the marginals. The displayed ellipses are close to degeneracy, i.e., θ≅π (left) and θ≅2π (right), where the method of ellipse fitting has known limitations. Especially large baseline fluctuations lead to large biases of the estimated phase close to these degeneracy points. Generally, the rotation angle α encodes the population ratio of the two involved states and reflects any imbalance due to imperfect state preparation. In addition, for any recorded bivariate dataset, histograms can be constructed and analyzed along a chosen direction α, complementing the ellipse-based analysis. For a detailed analysis of different choices of α, we refer to the Supplementary Materials but report here the key insights. From simple Gaussian uncertainty propagation, one expects that $S_{\text{sum}}$ is the optimal choice. However, this idea neglects a possible α dependence of the amplitude uncertainty arising from the explicit fitting procedure. Taking this into account shows that PEAC can benefit from a slight deviation from α=π/4 in terms of a better trueness over a wider range. The optimal choice of α can generally depend on the specific use case and application, such that we refrain to specify a single best value here and focus on α=π/4 for the remainder. In the following section, we investigate the systematic error and compare PEAC in trueness and precision to the ellipse fitting approach.

### PEAC performance

**Fig. 5.**
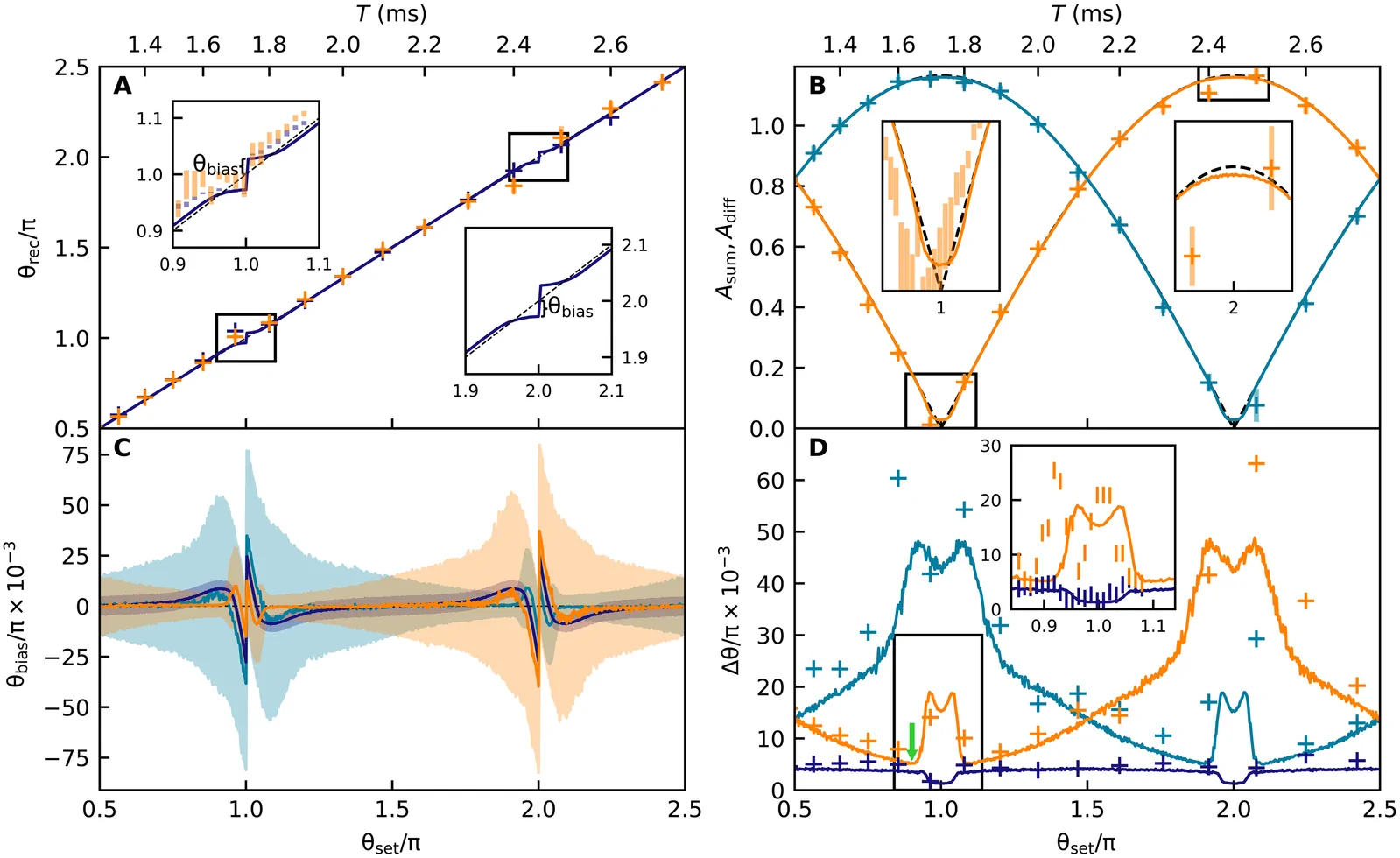
Performance of phase estimation: Ellipse fitting versus PEAC. Comparison of ellipse fitting (dark blue) and PEAC along the $S_{\text{sum}}$ (orange) and $S_{\text{diff}}$ (light blue) directions. Experimental data are shown as crosses (error bars omitted for clarity) or rectangular bars, with centers indicating the mean and heights equal to twice the bootstrapped standard deviation. Solid lines are values obtained from numerical replications of the experiment. The dashed lines correspond to the set phases and amplitudes as reference values free of fitting errors. We convert the set phase $\theta_{\text{set}}$ via Eq. 2 to T. (**A**) Reconstructed phase. The inset shows that ellipse fitting leads to a large bias $\theta_{\text{bias}}=\theta_{\text{rec}}-\theta_{\text{set}}$ near degeneracy despite high precision, while PEAC along the minor axis $S_{\text{sum}}$ exhibits larger uncertainty but a reduced bias. (**B**) Modulation of the amplitudes $A_{\text{sum}}$ and $A_{\text{diff}}$ obtained from histogram fits along the two principal axes. We observe a deviation at degeneracy, contributing to PEAC’s remaining bias. (**C**) Bias obtained from numerical replications. PEAC along the favorable (minor) axis increases the effective bias-free region by a factor of 1.6 compared to ellipse fits, substantially enhancing trueness. The semi-transparent bands give the uncertainties extracted from 1000 replicated experiments. (**D**) Precision of phase estimation. Ellipse fitting maintains low uncertainty across all $\theta_{\text{set}}$, while PEAC of $S_{\text{sum}}$ achieves comparable precision only near, but not at the degeneracy point $\theta_{\text{set}}=\pi$, as would be expected from simple Gaussian uncertainty propagation. This behavior arises from an ambiguity in the histogram’s PDF for small amplitudes. For PEAC, a distinct minimal uncertainty can be found, marking an optimal working point (see green arrow). The visible reduction in the ellipse’s uncertainty around the degeneracy points stems from the least-squares fitting routine converging to the boundary condition for the major semi-axis.

To quantify the performance of PEAC, we assess the trueness and precision of phase estimation, without relying on scanning the differential phase. Figure 5A displays the unwrapped phase $\theta_{\text{rec}}$, reconstructed individually from the experimental data for each T from geometric ellipse fitting (dark blue crosses, masked by orange crosses for most data points); see Materials and Methods for details. For a complementary theoretical analysis establishing a connection between experiment and theory, we use the control variable $\theta_{\text{set}}$, which we convert to the corresponding T via Eq. 2 using $a_{\text{ext}}=32.2(1)\;\text{mm}/s^{2}$. Ideally, we expect the black dashed line representing identity, corresponding to perfect phase reconstruction $\theta_{\text{rec}}=\theta_{\text{set}}$, yet notable deviations of the experimental data occur. This discrepancy becomes more evident for the numerical replication of the experiment, shown as the dark blue line. We use the experimentally inferred parameters characterizing the signals (see details in Materials and Methods). In essence, the numerical replication allows for a much finer sampling of $\theta_{\text{set}}$. The largest discrepancies occur at $\theta_{\text{set}}=\pi \mathbb{Z}$, i.e., at points of degeneracy, which are a fundamental limitation to ellipse-based phase estimation (*33*, *81*). We experimentally study one of these degeneracy points in more detail: The inset at $\theta_{\text{set}}=\pi$ illustrates this discrepancy through a finer experimental sampling in T. In this inset, the center of each rectangle denotes the mean, while its height is twice the empirical standard deviation extracted from bootstrapping, caused by the statistical nature of the baseline fluctuations. The experimental data obtained from ellipse fitting (dark blue) exhibits the same qualitative behavior as the numerical replication, thereby confirming the validity of our theoretical model. Hence, a bias $\theta_{\text{bias}}=\theta_{\text{rec}}-\theta_{\text{set}}$ in the vicinity of the degeneracy points deteriorates the trueness of the estimated phase.

Figure 5B presents the amplitudes $A_{\text{sum}}$ (orange) and $A_{\text{diff}}$ (blue) of $S_{\text{sum}}$ and $S_{\text{diff}}$, extracted from histogram fits. The data underline the collapse and revival behavior. Fits to the numerical replication lead to the solid lines for $A_{\text{sum}}$ (orange) and $A_{\text{diff}}$ (blue), which are phase shifted by π, as expected from Fig. 4. The insets magnify the deviation near the degeneracy points. As in Fig. 5A, we present the ideal amplitudes $A_{\text{sum}}=\sqrt{2}A_{0}\left|\text{cos}\left(\theta_{\text{set}}/2\right)\right|$ and $A_{\text{diff}}=\sqrt{2}A_{0}\left|\text{sin}\left(\theta_{\text{set}}/2\right)\right|$ by black dashed lines. Applying PEAC to the experimental data, we infer $\theta_{\text{rec}}\left(T\right)$ via Eq. 5 and thus obtain the orange crosses in Fig. 5A and rectangles in its inset. See Materials and Methods for a detailed description of the full protocol. Compared to the reconstruction via the ellipse method, we see a larger standard deviation and by that a decrease in precision.

The numerical replication allows us to quantify the respective bias $\theta_{\text{bias}}$ of the different estimation methods, shown in Fig. 5C as solid lines (dark blue: ellipse, orange: $S_{\text{sum}}$, blue: $S_{\text{diff}}$). Enabled by state-selective detection through the SG method and postprocessing of the data, the difference signal $S_{\text{diff}}$ is also analyzed using PEAC, revealing that the bias is π-phase shifted relative to $S_{\text{sum}}$. The traces directly show the averaged bias and are a measure of trueness (high if $\theta_{\text{bias}}≅0$), while the shaded areas represent the uncertainty ±Δθ in phase reconstruction, obtained from statistical repetitions as detailed in Materials and Methods, and quantify the precision: Smaller uncertainty bands correspond to higher precision. This plot illustrates the challenges of the ellipse fitting method: While it can achieve high precision, it suffers from major bias leading to low trueness, particularly at points of degeneracy. For our data, the ellipse fitting method has negligible bias except for an interval of ±π/4 around the degeneracy points. In contrast, PEAC substantially increases the bias-free region, so that a bias is only observed in an interval ±π/10 around degeneracy and therefore enhances the trueness at the expense of precision. We also observe that $S_{\text{sum}}$ and $S_{\text{diff}}$ reverse their roles at opposing degeneracy points, such that applying PEAC to $S_{\text{sum}}$ around θ≅π benefits from higher precision, while analyzing $S_{\text{diff}}$ is favorable around θ≅2π. Considering the bivariate $\left(S_{\text{sum}},S_{\text{diff}}\right)$ plane of Fig. 4, we understand this behavior geometrically: The bias and precision are only substantially reduced when the histogram is obtained by projecting along the minor axis of the ellipse. We give more insights into the bias’s origin in Materials and Methods. Along the favorable minor axis, PEAC extends the range of phases for which effectively bias-free estimation is possible by a factor of 1.6 compared to ellipse fitting. Even along the major axis, PEAC performs slightly better than ellipse fitting, although with a high uncertainty, which depends on the number of measuring points and further optimization schemes (*54*). For phases within ±π/10 around degeneracy, we observe considerable variations in bias across all three cases. Since the variation amplitude is the smallest for the favorable direction of PEAC, it maintains a reduced bias and remains the optimal choice for phase estimation in this regime. Thus, PEAC achieves higher trueness compared to the ellipse method, which is a necessary condition for high-accuracy measurements, for which both trueness and precision are essential.

Last, we compare the precision of both methods by investigating the phase uncertainty Δθ shown as shaded areas in Fig. 5C. The values of the uncertainty are displayed as solid lines in Fig. 5D for a direct comparison. To benchmark the theoretical analysis, we also add the corresponding experimental values as crosses and vertical bars in the inset around degeneracy (dark blue: ellipse fitting, orange: $S_{\text{sum}}$, blue: $S_{\text{diff}}$). The uncertainty of the ellipse method is almost constant across all phases and consistently lower than that of PEAC, for both $S_{\text{sum}}$ and $S_{\text{diff}}$. The dip of its uncertainty around degeneracy arises because the geometric ellipse fits are constrained by boundaries that enforce physically reasonable, i.e., normalized, principal axes. Apparently, the tendency to converge to these boundaries reduces the statistical uncertainty. Nevertheless, PEAC can achieve uncertainty levels as low as ellipse fitting for specific phases near, but not exactly at degeneracy. In quantum clock interferometry, simple Gaussian uncertainty propagation suggests $\Delta \theta =\Delta A_{\text{sum}}/\left|\partial_{\theta }A_{\text{sum}}\right|$, where $\Delta A_{\text{sum}}$ is the uncertainty of the oscillation amplitude. Following this intuition, the amplitude $A_{\text{sum}}=\sqrt{2}A_{0}\left|\text{cos}\left(\theta /2\right)\right|$ exhibits its steepest slope and by that the lowest uncertainty at θ=π. In contrast, a vanishing slope at θ=2π implies a diverging phase uncertainty and explains why the major axis is unfavorable for PEAC along $S_{\text{sum}}$. However, as shown in the inset of Fig. 5B, we observe that near the degeneracy point $\theta_{\text{set}}≅\pi$, the fitted value of $A_{\text{sum}}$ deviates from the expected behavior. This deviation and the connected increase in uncertainty arise from a merging of the two maxima of the PDF for low amplitudes, resulting in a single-peak PDF. Although no closed analytical expression describes this transition, a numerical analysis reveals that it occurs when A/σ<1.78, where σ is the standard deviation of the baseline fluctuations. Since A depends on θ, this transition from a double-peaked to a single-peaked PDF can be associated with a differential phase, which for our experimental parameters is 0.94π. This differential phase aligns well with our observation of an increase in both bias and uncertainty. To show that this observation holds for various settings, we performed a scan of the baseline noise σ. The corresponding two-dimensional scans are summarized and compared against ellipses in the Supplementary Materials. We observe that both bias and uncertainty of the phase estimation based on PEAC depend on baseline fluctuations. While ellipses show a superior behavior for vanishing noise, PEAC outperforms ellipse fits at higher noise levels. We observe that substantial bias is confined to a narrow region around degeneracy, where A<1.78σ, and increases linearly with σ. In this region, the increased ambiguity in the fit parameters $A_{\text{sum}}$ and σ results in larger bias $\theta_{\text{bias}}$ and increased phase uncertainty Δθ. Therefore, despite the expectation from Gaussian uncertainty propagation, the minimal phase uncertainty is not observed at the degeneracy point $\theta_{\text{set}}=\pi$, i.e., at vanishing signal amplitude. Minimal uncertainty is reached near, but not at the aforementioned degeneracy point, namely, where the signal amplitude is small but still finite. These insights allow us to define the working point to be that phase with no substantial bias and lowest phase uncertainty (see Fig. 5D green arrow). From our baseline-noise scan, we infer that the working point is in close proximity to the transition from a single-peak to a double-peak PDF. At this point, the level of uncertainty is similar to that of ellipse fitting, but the trueness is superior for nonvanishing baseline noise. Our analysis in the Supplementary Materials further indicates that this working point exhibits a linear scaling with σ. In addition and to establish a robust criterion for when an estimate can be trusted, we also normalize the bias in units of uncertainty in the Supplementary Materials, demonstrating that PEAC remains reliable throughout the entire interval in contrast to ellipses.

Naturally, these results depend not only on the specific noise and noise model but also on the chosen method of parameter estimation from a given PDF. In this article, we use a standard least-squares-based fitting routine because of its simplicity and numerical performance. However, other methods such as maximum likelihood estimation can provide better results. To compare these two approaches, we perform an analysis of bias and uncertainty in analogy to Fig. 5 (C and D) based on maximum likelihood estimation in the Supplementary Materials. In summary, we observe that this approach reduces both bias and uncertainty even further, albeit at the cost of a substantial increase in computational time. Given this trade-off, we have used least-squares-based fitting for our primary evaluation. However, the Supplementary Materials demonstrates that PEAC is inherently compatible with more sophisticated inference techniques and therefore compatible with further future improvements.

## DISCUSSION

Although the statistical analysis of the noisy signal of an interferometer, such as histogram-based estimation of the underlying PDF, provides access to the oscillation amplitude and baseline fluctuations, it inherently lacks phase information. When two such signals are correlated, common-mode noise is suppressed, allowing for the extraction of a differential phase θ from the bivariate data, e.g., from fitting to ellipses (*33*–*35*, *78*–*81*) or Lissajous curves (*17*, *85*).

In this work, we establish a geometric connection between these two paradigms: We introduce PEAC, a method that performs a statistical analysis along a suitably chosen projection direction in the bivariate plane. It extracts a differential phase from the observed amplitude, which shows a characteristic collapse and revival due to the beat of overlaying signals. Unlike conventional ellipse fitting, which becomes ill-posed near degeneracy points (θ=πℤ) where the ellipse collapses to a noisy line (*81*), PEAC always applies a statistical inference technique to the marginal distribution. This procedure eliminates the issues of a geometric degeneracy and reduces systematic bias, thereby enhancing trueness and overall accuracy. We highlight five key advancements:

1) PEAC allows extracting differential phases and amplitudes in setups where the individual signals cannot be resolved independently, e.g., in nonstate-selective setups where the individual components overlap spatially, overcoming one limitation of conventional ellipse fitting. While we demonstrated PEAC by scanning the differential phase in the “PEAC methodology” section to characterize the signal, such a scan is not a mandatory requirement. Provided that the system’s relevant parameters, such as the weights $\lambda_{m_{F}}$ and the intrinsic amplitude $A_{0}$, are determined through independent measurements or initial calibrations, the differential phase can be inferred without an explicit scan. This flexibility is particularly advantageous in tests of fundamental physics or quantum clock interferometry, where the measurand may be minute and modulation of the differential phase is often experimentally impractical.

2) It is a complementary analysis applicable to any system employing ellipse fitting without the need for additional experimental resources. While it generally requires more data points than ellipse fitting due to its statistical nature, PEAC becomes a powerful tool when sufficient statistics are available. It generalizes naturally by projecting along an angle α in the bivariate plane, enabling phase estimation of arbitrary mixtures of the correlated signals. For a given set of bivariate data, one can always perform PEAC along a different angle in addition to ellipse fitting (see the “Correlation measurements—Connection to ellipse fitting” section in combination with the Supplementary Materials) to improve the trueness of the estimated phase by reducing the bias and systematic error near degeneracy. While ellipse-based estimation is typically only unbiased at circular degeneracy points (θ=π/2 and odd multiples), PEAC provides a vanishing bias throughout the 2π interval of θ, except for a region of A<1.78σ around degeneracy. Consequently, PEAC is beneficial when the differential phase cannot be precisely controlled. Furthermore, in the “Correlation measurements—Connection to ellipse fitting” section, all parameters of the PDF were determined from independent measurements, experimentally demonstrating that differential phase estimation is possible without the necessity of controlling or scanning the measurand. A reduced bias closer to degeneracy is not merely a technical improvement but critical for high-accuracy null measurements, e.g., envisioned for tests of fundamental physics (*7*–*10*).

3) We demonstrate that PEAC directly connects to quantum clock interferometry (*39*–*44*) and answers the question of the optimal working point, which in fact is close to, but not exactly at, degeneracy. Notably, we observe no divergence of phase sensitivity close to degeneracy, as predicted from geometric phase amplification (*51*) when neglecting the vanishing oscillation amplitude or from Gaussian uncertainty propagation. Even in the context of fundamental physics applications, where one typically probes null signals or minute phase shifts and therefore has limited flexibility to access regimes with substantial amplitude collapse, PEAC has practical implications, as outlined above. Furthermore, recent quantum clock experiments using magnetic field gradients to measure geometric phases (*51*) have observed a considerable reduction in contrast, highlighting the relevance of such methods in realistic scenarios.

4) Beyond two correlated signals, we demonstrate the applicability of PEAC to three correlated signals, without requiring to resolve them individually, by the example of a mixture of three magnetic sublevels. The technique is therefore established as a complementary analysis of three- or higher-dimensional ellipse methods (*76*) by analyzing histograms along the semi-axes. Moreover, it can be applied to scenarios where an amplitude modulation is introduced deliberately into only one signal, situations that are inaccessible to ellipse fitting. For example, we extend contrast-envelope fits (*58*–*60*), a standard technique for atom interferometry, where the interferometer is partially or dynamically opened to control the visibility of the interference pattern. Similarly, it applies to schemes where a phase modulation translates into a characteristic amplitude modulation (*86*).

5) Last, PEAC is broadly applicable to a wide range of setups, from fully phase stable to noisy, phase-unstable regimes. The sensitivity of phase estimation can be enhanced by resorting to higher-order atom diffraction, demonstrating that it scales with increasing momentum transfer and can be applied to periodic signals beyond atom interferometry, such as atomic clocks (*55*, *56*) or optical interferometers. Moreover, the statistical nature of the method offers potential for integration into hybrid classical-quantum sensors (*87*) or for locking devices to a regime of minimal phase uncertainty (*88*).

We identified a working point for PEAC at phases close to, but not exactly at, degeneracy. Using a numerical model and associated parameter scans, we showed that its exact location depends approximately linearly on the strength of baseline fluctuations. However, although a preferred phase of operation exists, PEAC remains applicable even when the differential phase cannot be sufficiently controlled. In this case, ellipse fitting exhibits a substantial bias across all phases except at θ=π/2, exceeding the statistical uncertainty, whereas PEAC yields a bias-free estimate except in a narrow region around degeneracy. By contrast, in the idealized absence of baseline fluctuations, ellipse fitting outperforms PEAC, as demonstrated by our numerical analysis.

Moving beyond the least-squares fitting of PDFs used in the main text, PEAC can be further improved by combining it with alternative statistical inference techniques, such as maximum likelihood estimation (*89*) discussed in the Supplementary Materials. Furthermore, it can be integrated with other approaches such as Bayesian methods (*17*, *54*, *90*). As these techniques pose a more targeted approach to parameter estimation, we anticipate a further reduction of the bias. Similarly, PEAC can be combined with recent developments in ellipse fitting, such as geometric procedures that minimize the bias with the help of optimized cost functions (*21*). Using PEAC in combination with ellipse fitting and inducing a feedback loop between these procedures can help to decrease the bias in a joint phase estimation protocol, especially close to degeneracy. One could further allow for adaptive bin sizes within a single histogram and automatically assess the number of required bins to resolve the important features of the PDF without increasing the statistics. Such a procedure would also lead to improved initial guesses for the fitting routines. PEAC is a classical statistical method, and the presented experimental results are obtained in a setup limited by technical noise one order of magnitude above shot noise. Further reducing technical noise sources leads to a regime in which the sensitivity is limited by quantum projection noise, i.e., shot noise. In this case, the signal is no longer homoscedastic, and PEAC must be adapted accordingly. At the same time, this regime constitutes the prerequisite for quantum-enhanced metrology. Combining PEAC with quantum input states, such as optimally squeezed states (*84*), has the potential to surpass current limits in quantum metrology and to advance perspectives in a broad range of applications.

## MATERIALS AND METHODS

### Experimental system and measurements

Our experimental setup produces BECs in a CDT by forced evaporative cooling (*62*, *91*). To perform measurements in free space, the atoms are released from the CDT into free fall. Prior to the start of the MZI sequence, an expansion time of 3 ms allows for the conversion of the mean-field to kinetic energy. After this expansion, typically (*63*) the momentum spread of the ensemble is $\Delta p=0.13\left(3\right)\;\hbar k_{\text{eff}}$. The laser beams used for Bragg diffraction are superimposed onto one of the CDT beams in a counter-propagating setup, oriented perpendicular to the direction of gravity. This ensures an initial momentum of $p_{0}=0$ in the direction of Bragg diffraction. To drive two-photon transitions, the Bragg beams are detuned by Δ=2π×3.2 GHz from the excited state manifold $|e\rangle =|5^{2}P_{3/2},F=2\rangle$, transferring momentum $\hbar k_{\text{eff}}=2\hbar \times 2\pi /780.226\;\text{nm}$ along the Bragg axis. For the results presented in this article, we rely on first-order Bragg diffraction by choosing a detuning Δω=2π×15.084 kHz between the two beams, while our setup also allows for higher-order diffraction (*63*) by adjusting Δω to other resonances. A Blackman window function f(t)=0.42−0.5cos(2πt/τ)+0.08cos(4πt/τ) for 0≤t≤τ as a smooth pulse shape ensures efficient transfer between the coupled momentum states and reduces the population of parasitic momenta. We use a pulse length of τ=100 μs as a compromise between velocity selectivity and diffraction efficiency, adjusting the peak Rabi frequency of the three pulses to adapt for the π/2−π−π/2 pulse configuration. Following each MZI, a TOF of typically 15 to 30 ms is applied to separate the two interferometer ports spatially for detection. We perform resonant absorption imaging (*92*) of the atomic densities to gain access to the atom numbers $N_{0}$ and $N_{1}$ in each of the interferometer ports. Without optical pumping or a state-selective SG measurement, the densities of the three $m_{F}$ substates will spatially overlap and cannot be distinguished. However, our setup also allows using the SG method by applying a magnetic field gradient after the MZI, generated by one of the magnetic coils used for the magneto-optical trap, separating the $m_{F}$ substates spatially via the Zeeman force. A time delay of 1 ms between the second π/2 pulse and the introduction of the magnetic field gradient prevents spurious phase contributions to the interferometer phase. The applied weak gradient is left on for a total of 24 ms until detection to ensure proper state separation.

### Derivation of signal *S*

The normalized difference between the atom numbers $N_{j}\left(\phi \right)=B_{j}\pm A_{j}\text{cos}\phi$ with j∈{0,1} detected in the two momentum classes leads to the signal

$$S=\frac{N_{0}-N_{1}}{N_{0}+N_{1}}=\frac{\Delta B+2\bar{A}\text{cos}\phi }{2\bar{B}+\Delta A\text{cos}\phi }$$ (7)

introducing the mean and differential baseline $\bar{B}=\left(B_{0}+B_{1}\right)/2$ and $\Delta B=B_{0}-B_{1}$, as well as the mean and differential amplitude $\bar{A}=\left(A_{0}+A_{1}\right)/2$ and $\Delta A=A_{0}-A_{1}$, respectively. With only two exit ports, the total atom number $N_{0}+N_{1}$ must be preserved and independent of the interferometric phase ϕ, implying $A_{1}=A_{0}$. As a result, we find

$$S=\frac{\Delta B}{2\bar{B}}+\frac{A_{0}}{\bar{B}}\text{cos}\phi$$ (8)

In general, $A_{j},B_{j}$ are suitable random variables describing experimental stochastic fluctuations of baselines and amplitudes, which may cause a heteroscedastic signal. It is typically assumed that the baseline noise $\Delta B/\left(2\bar{B}\right)$ is normally distributed and dominant, such that $A_{0}/\bar{B}$ can be treated as constant (*54*). Observing a homoscedastic signal, i.e., in our setting treating $A_{0}/\bar{B}$ as constant, is possible if both random variables are correlated. Assuming that $A_{0}$ and $\bar{B}$ are perfectly correlated gives

$$A_{0}=\sqrt{\frac{V\left[A_{0}\right]}{V\left[\bar{B}\right]}}\left(\bar{B}-E\left[\bar{B}\right]\right)+E\left[A_{0}\right]$$ (9)

where E[⋅] denotes the expectation value and V[⋅] the variance of a random variable. To have constant $A_{0}/\bar{B}$ and excluding the trivial constant signal, i.e., $E\left[A_{0}\right]=0$, the condition

$$\frac{E\left[A_{0}\right]}{E\left[\bar{B}\right]}=\sqrt{\frac{V\left[A_{0}\right]}{V\left[\bar{B}\right]}}$$ (10)

must be satisfied. With Eq. 10, we observe from the condition of perfect correlation (*9*) that the signal takes the form

$$S=\frac{\Delta B}{2\bar{B}}+\frac{E\left[A_{0}\right]}{E\left[\bar{B}\right]}\text{cos}\phi$$ (11)

in which only $\Delta B\;/\left(2\bar{B}\right)$ fluctuates.

Assuming normally distributed baselines $B_{j}∼N\left(\mu_{j},\sigma_{j}^{2}\right)$ with mean $\mu_{j}$ and standard deviation $\sigma_{j}$, for resolving a signal, the fluctuations must be sufficiently small, i.e., $\mu_{j}\gg \sigma_{j}$. If we additionally assume the same baseline fluctuations in both exits of the interferometer, i.e., $\sigma_{1}=\sigma_{2}$, then we can treat the mean baseline as constant $\bar{B}∼N\left(\bar{\mu },\sigma_{1}^{2}/2\right)≅\bar{\mu }=E\left[\bar{B}\right]$ with $\bar{\mu }=\left(\mu_{1}+\mu_{2}\right)/2$ and $\sigma_{1}=\sigma_{2}\ll \bar{\mu }$. With these stipulations we finally arrive at a homoscedastic signal

S=B+Acosϕ (12)

with $B=\Delta B\;/\left(2E\left[\bar{B}\right]\right)∼N\left(\mu ,\sigma^{2}\right)$ of mean $\mu =\left(\mu_{1}-\mu_{2}\right)/\left(2\bar{\mu }\right)$ and variance $\sigma^{2}=\sigma_{1}^{2}/\left(2{\bar{\mu }}^{2}\right)$, and $A=E\left[A_{0}\right]/E\left[\bar{B}\right]$, that has no amplitude fluctuations and only normally distributed baseline fluctuations.

### Finite-pulse duration

The finite duration τ of the Bragg pulses can be a substantial fraction of the interferometer time T, ranging from 3% up to 10% in our experiments and therefore introduces a non-negligible phase contribution (*68*, *93*–*95*) that must be included in our phase analysis. To incorporate this phase contribution, we resort to a numerical treatment, necessary for time-dependent pulses and define the accumulated time-dependent pulse area

$$\phi_{1}\left(t\right)=\int_{0}^{t}dt^{\prime }\;\Omega \left(t^{\prime }\right)$$ (13)

with the time-dependent Rabi frequency train $\Omega (t)=\Omega_{0}\left[f(t)+2f\left(t-T\right)+f\left(t-2T\right)\right]$ described by the Blackman envelope f. As in the experiment, we choose $0.42\;\Omega_{0}\tau =\pi /2$ accounting for the π/2 pulses at t=0 and t=2T and for the π pulse at t=T. An external acceleration $a_{\text{eff}}$ induces a time-dependent Doppler detuning $k_{\text{eff}}a_{\text{eff}}t$, assuming that the atoms are initially on resonance in agreement with our experimental realization. For example, to obtain the phase of the $m_{F}=+1$ state induced by $a_{\text{eff}}$, we evaluate (*68*)

$$\theta \left(T\right)/2=\int_{0}^{2T+\tau }dt\;k_{\text{eff}}a_{\text{ext}}t\;\text{sin}\left[\phi_{1}\left(t\right)\right]$$ (14)

numerically for τ=100 μs and various T∈[1 ms,3 ms]. For a simplified analytical expression, we fit $\theta \left(T\right)=2k_{\text{eff}}a_{\text{eff}}T^{2}\left(1+\gamma \tau /T\right)$ to the resulting values and obtain the fit parameter γ≅0.1486.

### General amplitude modulation

Here, we present the derivation of the amplitude modulation $A_{\text{all}}$ in Eq. 3 of the main text. A sum of n cosine functions weighted by $\lambda_{i}\in \mathbb{R}$ with a common phase $\phi_{0}$, but possible different phases $\theta_{i}$, results (*96*) in

$$\sum_{i=1}^{n}\lambda_{i}\text{cos}\left(\phi_{0}+\theta_{i}\right)=A\left[\{\lambda_{i}\},\{\theta_{i}\}\right]\text{cos}\left(\phi_{0}+\theta_{\text{off}}\right)$$ (15a)

with the modulated amplitude

$$A^{2}=\sum_{i=1}^{n}\lambda_{i}^{2}+2\sum_{i=1}^{n}\sum_{j>i}^{n}\lambda_{i}\lambda_{j}\text{cos}\left(\theta_{i}-\theta_{j}\right)$$ (15b)

and offset phase

$$\text{tan}\theta_{\text{off}}=\frac{\sum_{i=1}^{n}\lambda_{i}\text{sin}\theta_{i}}{\sum_{i=1}^{n}\lambda_{i}\text{cos}\theta_{i}}$$ (15c)

In our setting we have $\left\{\lambda_{i}\right\}=\left\{\lambda_{m_{F}}\right\}$, $\theta_{0}=0$, and $\theta_{\pm 1}=\pm \theta /2$, such that after algebraic manipulations, we obtain Eq. 3 of the main text and find for the offset phase

$$\text{tan}\theta_{\text{off}}=\frac{\lambda_{0}+\Delta \lambda \text{sin}\left(\theta /2\right)}{\lambda_{0}+\left(\lambda_{-1}+\lambda_{+1}\right)\text{cos}\left(\theta /2\right)}$$ (16)

A pointwise phase reconstruction is possible if all $\lambda_{m_{F}}$ are known and can be obtained from

$$\text{cos}\left(\theta /2\right)=\frac{\lambda_{0}\left(\Lambda -\lambda_{0}\right)}{\Delta \lambda^{2}-{\left(\Lambda -\lambda_{0}\right)}^{2}}\pm \sqrt{\frac{\Delta \lambda^{2}\left[\Delta \lambda^{2}+2\Lambda \lambda_{0}-\Lambda^{2}\right]}{{\left[\Delta \lambda^{2}-{\left(\Lambda -\lambda_{0}\right)}^{2}\right]}^{2}}-\frac{{\left(A_{\text{all}}/A_{0}\right)}^{2}}{\Delta \lambda^{2}-{\left(\Lambda -\lambda_{0}\right)}^{2}}}$$ (17)

where we introduced $\Lambda =\sum_{m_{F}}\lambda_{m_{F}}$.

### Histogram fitting routine

We generate all histograms by binning the 300 measurements of each signal into $\sqrt{300}≅18$ bins. The width of the bins for each T setting is adapted so that all 18 bins cover the whole range of acquired data and therefore depends on the signal’s maximum value. This change of bin size can be seen in Figs. 3B and 4. The square root rule is an established rule for creating histograms with an adequate resolution in our setting, although we note that this is not a trivial matter and leaves plenty of room for refinement (*97*, *98*).

#### Probability density function

We start from the signal form of the main text, i.e., $S=B+A\text{cos}(\phi_{0}+\theta_{\text{off}})$, where A is a constant amplitude, $\phi_{0}$ is a (potentially fluctuating) phase that is used to scan a fringe, $\theta_{\text{off}}$ is a fixed phase, and B is a normally distributed random variable describing baseline fluctuations.

At the same time, fluctuations of $\phi_{0}$ translate into a PDF for the observed amplitude (*99*)

$$f_{A}\left(a\right)=\left\{\begin{array}{cc} 1/\left(\pi \sqrt{A^{2}-a^{2}}\right) & ,\;-A<a<A\\ 0 & ,\;\text{else} \end{array}\right.$$ (18)

if one assumes a uniform phase distribution with support [0,2π). By linearly scanning $\phi_{0}$ in our experiment, we ensure that this uniform phase distribution is a good approximation, even in the absence of phase fluctuations, i.e., for short T. However, any underlying PDF of $\phi_{0}$ can be used to adapt $f_{A}$.

Moreover, we assume that baseline fluctuations are independent and identically distributed, such that their PDF is given by a normal distribution

$$f_{B}\left(b\right)=\frac{1}{\sigma \sqrt{2\pi }}\text{exp}\left[-\frac{{\left(b-\mu \right)}^{2}}{2\sigma^{2}}\right]$$ (19)

with mean μ and variance $\sigma^{2}$, according to the central limit theorem. The PDF of a sum of two random variables is the convolution ∗ of their respective PDFs, i.e., the PDF of the signal S is

$$f_{S}\left(s\right)=\left(f_{A}*f_{B}\right)\left(s\right)=\frac{1}{\sigma \sqrt{2\pi^{3}}}\int_{-A}^{A}da\;\frac{1}{\sqrt{A^{2}-a^{2}}}\text{exp}\left[-\frac{{\left(s-a-\mu \right)}^{2}}{2\sigma^{2}}\right]$$ (20)

For A≥2σ, this PDF shows a symmetric double-peak distribution. These maxima are roughly separated by 2A, their width is determined by σ, and the PDF rapidly decays for ∣s∣→∞. Contrary, for A<2σ, the two maxima start to overlap, causing the double-peak structure to become washed out.

#### Initial guesses

For fitting the histogram’s PDF, see Eq. 5, we use scipy.optimize.curve_fit from the scipy package in python (*100*), returning $A_{\text{fit}}$, $\sigma_{\text{fit}}$, and $\mu_{\text{fit}}$. To guarantee reliable convergence of the fit, accurate initial parameter guesses are essential. Note that the scipy.optimize.curve_fit method is based on a gradient approach, which is computationally efficient; however, the PDF $f_{S}$ is not differentiable with respect to A even in a distributional sense. We noticed issues related to this nondifferentiability when using poor initial guesses.

The initial guess of the histogram’s mean $\mu_{\text{guess}}$ is given by explicitly calculating the signal’s mean. A reliable initial guess for the standard deviation of the baseline fluctuations $\sigma_{\text{guess}}$ can be extracted from the rapid decay ∣s∣→∞ of the PDF: First, we determine one maximum of the histogram at $s_{\text{max}}$. Starting from this maximum, we scan outward across the histogram to locate the position $s_{k}=ks_{\text{max}}$ with 0<k<1, where the maximum has dropped to the fraction k. We then roughly approximate the rapid outward drop of the histogram by an exponential decay, resulting in

$$\sigma_{\text{guess}}=\left|s_{\text{max}}-s_{k}\right|\sqrt{-1/\left(2\text{ln}k\right)}$$ (21)

which is well-defined since lnk<0. The choice k=1/4 gave robust and reliable initial guesses.

The initial guess of the amplitude $A_{\text{guess}}$ is obtained from the above guesses via

$$A_{\text{guess}}=\left|s_{\text{max}}-\mu_{\text{guess}}\right|+\sigma_{\text{guess}}$$ (22)

We numerically confirmed that $s_{\text{max}}≅\mu \pm \left(A-\sigma \right)$, up to numerical factors. An analytical form is only possible for σ=0, where we find $s_{\text{max}}=\mu \pm A$ as expected.

#### Fitting procedure

When fitting a single histogram, we make use of the initial guesses according to the “Initial guesses” section. In the state-selective case, we have access to $S_{\pm 1}$, $S_{\text{sum}}$, and $S_{\text{diff}}$, such that we can perform four histogram fits. We start by fitting the histogram’s PDF to $S_{\pm 1}$ based on the initial guesses from the previous section, returning $A_{\pm 1,\text{fit}}$, $\sigma_{\pm 1,\text{fit}}$, and $\mu_{\pm 1,\text{fit}}$. We set $A_{0}$ to be the mean $\left(A_{+1,\text{fit}}+A_{-1,\text{fit}}\right)/2$. Next, we use the mean $\left(\sigma_{+1,\text{fit}}+\sigma_{-1,\text{fit}}\right)/2$ as the initial guess for $\sigma_{\text{sum},\text{fit}}$ and $\sigma_{\text{diff},\text{fit}}$, unless it is smaller than any bin width of $S_{\pm 1}$. In the latter case, we use the initial guess routine of the “Initial guesses” section for $\sigma_{\text{sum},\text{fit}}$ and $\sigma_{\text{diff},\text{fit}}$. Both $S_{\text{sum}}$ and $S_{\text{diff}}$ fluctuate with the same standard deviation σ, identical to that of $S_{\pm 1}$, because for $B_{i}∼N\left(\mu_{i},\sigma_{i}^{2}\right)$, the random variable $Z=\lambda_{1}B_{1}+\lambda_{2}B_{2}$ is also normally distributed, i.e., $Z∼N\left(\lambda_{1}\mu_{1}+\lambda_{2}\mu_{2},\lambda_{1}^{2}\sigma_{1}^{2}+\lambda_{2}^{2}\sigma_{2}^{2}\right)$. Since we have $\sigma_{1,2}=\sigma$ and $\lambda_{\pm 1}=1/\sqrt{2}$, we find $\lambda_{1}^{2}\sigma_{1}^{2}+\lambda_{2}^{2}\sigma_{2}^{2}=\sigma^{2}$.

Last, we restrict the parameter $A_{\text{sum},\text{fit}}$ to $\left[0,\sqrt{2}A_{0,\text{fit}}\right]$ but otherwise estimate the amplitudes as in the “Initial guesses” section. These limits ensure the correct connection between $S_{\pm 1}$ and $S_{\text{sum}}$, allowing for a phase reconstruction via Eq. 5.

We apply the same procedure to the difference signal, but use $S_{\text{diff}}=\sqrt{2}A_{0}\left|\text{sin}\left(\theta /2\right)\right|$ for phase reconstruction. Unlike the amplitude and standard deviation of the baseline fluctuations, the mean is not an issue. Therefore, we use the mean of the respective signal as the initial guess.

### Ellipse fitting routine

As a comparison to PEAC, we deploy an established approach to evaluate correlated signals based on fitting an ellipse to a bivariate parametric plot of two signals. We assume that the signals of the $m_{F}=\pm 1$ substates have the parametric form $S_{\pm 1}\left(\phi_{0}\right)=B_{\pm 1}+A_{\pm 1}\text{cos}\left(\phi_{0}\pm \theta /2\right)$ with amplitudes $A_{j}$, baselines $B_{j}$, common phase $\phi_{0}$, and differential phase θ. The bivariate data span ellipses in the $\left(S_{+1},S_{-1}\right)$ plane, where the eccentricity of the ellipse encodes the differential phase. We translate the parametric form into the algebraic description $0=c_{+1}^{2}S_{+1}^{2}+c_{-1}^{2}S_{-1}^{2}+c_{0}S_{+1}S_{-1}+d_{+1,-1}S_{+1}+d_{-1,+1}S_{-1}+d_{0}$ of conic sections with the coefficients

$$\begin{array}{c} c_{j}={\left(A_{j}\text{sin}\theta \right)}^{-1}\;\text{and}\;c_{0}=-2c_{+1}c_{-1}\text{cos}\theta \\ d_{i,j}=-2B_{i}c_{i}^{2}-B_{j}c_{0}\;\text{and}\;d_{0}=B_{+1}^{2}c_{+1}^{2}+B_{-1}^{2}c_{-1}^{2}+B_{+1}B_{-1}c_{0}-1 \end{array}$$ (23)

which for ellipses have to satisfy $c_{0}^{2}-4c_{+1}^{2}c_{-1}^{2}=-4c_{+1}^{2}c_{-1}^{2}{\text{sin}}^{2}\theta <0$ for θ∈(0,π)+πℤ. For θ=πℤ the ellipse degenerates onto straight lines in the case of perfect correlation or anti-correlation. The direct evaluation of Eq. 8 gives access to the differential phase

$$\theta =\pm \text{arccos}\left(-c_{0}/\sqrt{4c_{+1}^{2}c_{-1}^{2}}\right)+\pi \mathbb{Z}$$ (24)

As mentioned in the main text, diagonalizing the quadratic form $c_{+1}^{2}S_{+1}^{2}+c_{0}S_{+1}S_{-1}+c_{-1}^{2}S_{-1}^{2}$ and assuming equal amplitudes $A_{+1}=A_{-1}$ implying $c_{+1}=c_{-1}$ leads to the two principal axes of the ellipse along the ${\left[1,\text{sgn}\left(c_{0}\right)\right]}^{T}/\sqrt{2}$ and ${\left[1,-\text{sgn}\left(c_{0}\right)\right]}^{T}/\sqrt{2}$ direction of the bivariate coordinate space, i.e., the diagonal and antidiagonal depending on the sign of $c_{0}$.

Expressed in the bivariate plane, the signal for $A_{\pm 1}=A_{0}$ is

$$\left(\begin{array}{c} S_{-1}\\ S_{+1} \end{array}\right)=\left(\begin{array}{c} B_{-1}\\ B_{+1} \end{array}\right)+A_{0}\text{cos}\frac{\theta }{2}\left(\begin{array}{c} 1\\ 1 \end{array}\right)\text{cos}\phi_{0}+A_{0}\text{sin}\frac{\theta }{2}\left(\begin{array}{c} 1\\ -1 \end{array}\right)\text{sin}\phi_{0}$$ (25)

Clearly, this equation describes an ellipse with semi-axes along the diagonal/antidiagonal of the bivariate plane. Hence, we perform a geometric fit to general form

$$\left(\begin{array}{c} S_{-1}\\ S_{+1} \end{array}\right)=\left(\begin{array}{c} B_{-1}\\ B_{+1} \end{array}\right)+a\left(\begin{array}{c} \text{cos}\xi \\ \text{sin}\xi \end{array}\right)\text{cos}\phi_{0}+b\left(\begin{array}{c} -\text{sin}\xi \\ \text{cos}\xi \end{array}\right)\text{sin}\phi_{0}$$ (26)

where a,b are the moduli of the semi-axes and ξ is the ellipse’s rotation angle in the bivariate plane. Using Eq. 15 to cast this into the form of Eq. 25 gives

$$\theta =\text{arctan}\left(\frac{b\text{sin}\xi }{a\text{cos}\xi }\right)-\text{arctan}\left(\frac{-b\text{cos}\xi }{a\text{sin}\xi }\right)$$ (27)

This form of the differential phase θ allows using the two-argument arctangent of many programming languages. Minimizing the sum of the squared Euclidean distance gives rise to the so-called geometric ellipse fits (*81*, *82*, *84*). We use scipy.optimize.least_squares and scipy.optimize.minimize_scalar for this minimization process with the physical bounds $B_{\pm 1}\in \left[-1,1\right]$, $a,b\in \left[0,\sqrt{2}\right]$, and ξ∈[−π,π] as we work with normalized signals. Because of its computational low cost, we use the Halíř and Flusser algorithm (*79*, *80*) to fit the algebraic ellipse Eq. 23 to our data as an initial guess for the computationally more demanding geometric ellipse fits.

### Bootstrapping

We assume that the experimental uncertainties of the homoscedastic interferometric signals S stem from fluctuations of the signal’s baseline B, parametrized by the standard deviation σ of a normal distribution in our model, while the amplitude of the signal is assumed to be constant. Since for any interferometer time T we fit the PDF to all recorded data points, no uncertainty of this procedure can be extracted in a direct way. To estimate the uncertainties of measured quantities, we therefore implement a bootstrapping approach (*71*, *72*, *101*), assuming that our data provide a representative sample of the underlying probability distribution. For each interferometer time T, we use all 300 available data points and randomly draw a set of 300 data points with replacement. In addition to the original data, we generate 999 random sets, giving us access to 1000 independent datasets per T, each comprising 300 points. These datasets are then used to obtain the quantities of interest such as the amplitude A or the differential phase θ via least-square fitting methods. Based on the extracted values, we compute mean and standard deviations for each quantity, which are reported in the main body of this article.

To validate the reliability of the bootstrapping procedure, we compare it against randomly generated numerical datasets. We find no substantial difference in the standard deviations between random and bootstrapped datasets.

### Numerical replication

To investigate properties such as the bias $\theta_{\text{bias}}$ or the uncertainty Δθ with finer resolution than experimentally being accessible with reasonable effort, we resort to a numerical replication of our experiment. For the numerically generated data, we use the amplitude $A_{0,\text{exp}}=0.824$ and baseline fluctuations $\sigma_{\text{exp}}=0.063$ extracted from the experimental datasets, setting the mean baseline offset μ=0, since it corresponds to a translation that can be neglected. Similar to bootstrapping the experimental data, we produce 1000 numerical datasets containing 300 randomly generated values $S_{\pm 1}$ for each phase θ of interest. Here, we markedly increase the sampling of θ compared to the experiment to give access to finer details. Each random value of $S_{\pm 1}$ is computed by sampling a random phase $\phi_{0}\in \left[0,2\pi \right)$ and a baseline value $B_{\pm 1}$ drawn from a normal distribution with mean μ=0 and variance $\sigma_{\text{exp}}^{2}$. We implement the random number generation using the numpy.random.default_rng.uniform and numpy.random.default_rng.normal functions of the numpy package in python (*102*, *103*). By producing 1000 individual datasets, rather than bootstrapping a single set, as it was done with the experimental data, we can check whether a systematic effect is apparent in the bootstrapping procedure itself, which assumes that the experimental sample size is sufficient to describe the underlying PDF. In our case, we find no systematic difference between the bootstrapped experimental data and numerically generated random data, hence we observe no systematic effects.

### Origin of a bias in PEAC

We identified three main challenges that cause a bias in PEAC: (i) the signal’s amplitude A becomes comparable to the standard deviation of the baseline fluctuations σ; (ii) the true amplitude is close to the fit boundaries; and (iii) resolution limits imposed by bin size. In contrast to ellipse fits at the points of degeneracy, where the algebraic structure reduces to a line and fitting data to an ellipse is ill-posed, the underlying PDF used in PEAC is correct for all differential phases. Thus, the observed bias is not intrinsic to PEAC, but reflects limitations of any fit procedure in regimes of ambiguous parameters, parameters close to boundaries, or a finite resolution.

Addressing (i): Because of the amplitude modulation, the PDF of the histogram transitions from a double-peak to a single-peak distribution, as the two maxima begin to overlap until only one maximum remains, depending on the differential phase. Although no analytical form exists for this transition, we numerically found a threshold at A/σ≅1.7777. Below this limit, two equally plausible fits are possible because of the ambiguity in the combination of A and σ. This ambiguity leads to a wider spread in the amplitude values A, resulting in a wider spread of $\theta_{\text{rec}}$ and by that increasing its uncertainty Δθ. Consequently, the true amplitude value becomes less likely, resulting in a distorted distribution overestimating the mean. A maximum likelihood estimation (*89*) instead or a Bayesian inference strategy (*17*, *54*, *90*) could in principle be used to mitigate this issue.

Addressing (ii): To ensure consistency between $S_{\pm 1}$ and $S_{\text{sum}},S_{\text{diff}}$, we impose the fit limits $A_{\text{sum}},A_{\text{diff}}\in \left[0,\sqrt{2}A_{0}\right]$. For example, the upper limit guarantees that a phase reconstruction via Eq. 5 is possible, as higher amplitudes imply values outside the domain of the arccos function. However, this constrain leads to a truncated distribution of fit parameters, whose mean underestimates the true amplitude value. Over- and underestimating are general limitations of any fit routine with physical or logical bounds of the fit parameters.

Addressing (iii): We use a fixed number of 18 bins, but the range of signal values $S_{\text{sum}}$ and $S_{\text{diff}}$ changes with the differential phase θ because of the modulation of $A_{\text{sum}}$ and $A_{\text{diff}}$. A wider range of values implies bigger bin sizes reducing the fit resolution. In combination with (ii), this effect is most likely the reason for the bias in the difference signal (along the major axis) being bigger than the bias in the sum signal (along the minor axis) at θ=π.

## Supplementary Materials

### This PDF file includes:

Sections S1 to S5 Figs. S1 to S8
