# Supplementary material for: Parameter estimation from amplitude collapse in correlated matter-wave interference
Source: Sci Adv. 2026 Aug 26;12(35):eaeg5334. doi: 10.1126/sciadv.aeg5334 (PMC13510596; doi:10.1126/sciadv.aeg5334)
Supplement: Supplementary file 1 [file sciadv.aeg5334_sm.pdf]

Science Advances
AAAS

Supplementary Materials for

Parameter estimation from amplitude collapse in correlated matter-wave interference

Daniel Derr *et al.*

Corresponding author: Daniel Derr, daniel.derr@physik.tu-darmstadt.de, daniel.derr@gmx.net

Sci. Adv. **12**, eaeg5334 (2026)
DOI: 10.1126/sciadv.aeg5334

This PDF file includes:

Sections S1 to S5
Figs. S1 to S8

S1. GEOMETRIC VS. ALGEBRAIC ELLIPSE FITS

In the main body of the article, we compared PEAC to geometric ellipse fits, realised by a least-squares implementation, for more details see Materials and Methods. Since both signals S_{+1} and S_{-1} are normalised, the semi axes of the corresponding ellipses have to be confined to the interval $[0, \sqrt{2}]$, a physical constraint that we implemented in our fitting routine. To optimise the computational cost, we used the standard approach of fitting the algebraic form of the ellipse via the algebraic Halíř–Flusser algorithm [79, 80] and convert its output to the geometric form as an initial guess for geometric ellipse fitting. Naturally, it can be expected that geometric ellipse fits return a lower bias and uncertainty when compared to the algebraic form [81, 82]. To verify this established result, we compare both approaches with special emphasis on then degeneracy point $\theta = \pi$. Figure Fig. S1 shows bias (**A**) and uncertainty (**B**) for both algebraic (orange) and geometric (dark blue) ellipse fits in the interval $\pi/2 \leq \theta \leq 3\pi/2$. As expected, the geometric ellipse fits generate a lower bias over the whole interval with comparable uncertainty, and reduce it by a factor of approximately two at the degeneracy point $\theta = \pi$. Nevertheless, both fitting techniques are only bias free when the ellipse turns into a circle at $\theta = \pi/2$. Inside the region marked by dashed lines, where PEAC has an increased bias and uncertainty due to the ambiguity of fit parameters, geometric ellipse fits show a reduced uncertainty, as in this region the fitting routine trends towards the boundary condition $\sqrt{2}$ for the major half axes of the ellipse, effectively fixing one parameter at this value. Hence, the reduction in uncertainty is deceptive and does not provide an increase in accuracy, especially given the substantial bias in this region. In summary, we confirm the expected performance gain of geometric ellipse fits compared to the algebraic approach at the cost of increased computation time.

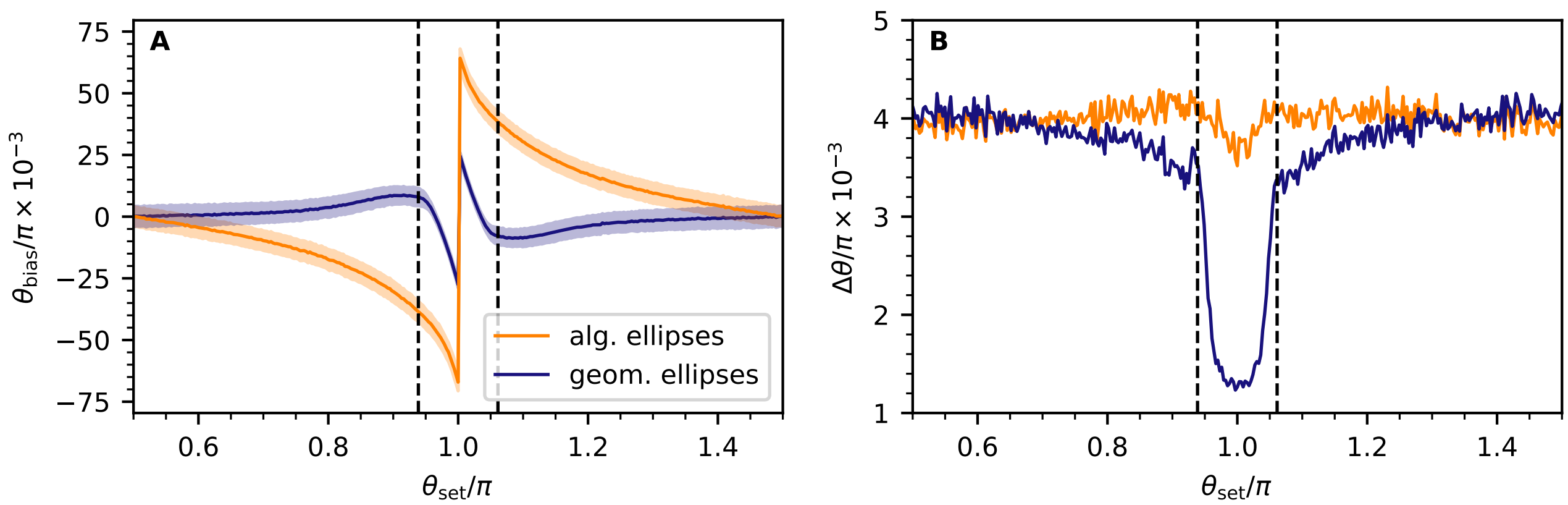

FIG. S1. **Comparison of geometric and algebraic ellipse fits.** (**A**) Fitting ellipses using the geometric description (dark blue) reduces the bias θ_{bias} compared to the algebraic fit (orange) based on the Halíř–Flusser algorithm [79, 80]. Nevertheless, independent of the fitting routine, ellipses become bias free only for $\theta = \pi/2$ and show an increased bias at the degeneracy point $\theta = \pi$. (**B**) In contrast to the bias, the uncertainty $\Delta\theta$ remains similar for both fitting routines outside the interval marked by dashed lines. The apparent reduction of the bias inside this region stems from the fit converging to the boundary of the major semi axis at $\sqrt{2}$, which effectively reduces the uncertainty.

S2. IMPACT OF BASELINE FLUCTUATIONS

To generalise the results of the main body of the article beyond situations tailored to our specific experimental parameters, we examine the impact of various baseline fluctuations. In analogy to the numerical replication of the experiment, we generate a set of random bivariate data scanning the differential phase $\theta_{\text{set}} \in [\pi/2, \pi]$ for different baseline fluctuations σ, and the intrinsic amplitude $A_0 = 0.824$.

We employ PEAC along S_{sum} and, for comparison, geometric ellipse fits to these numerical datasets. The results of these parameter scans are shown as density plots in Figs. S2 for PEAC and S3 for geometric ellipse fits, respectively. In both figures, panel (**A**) shows the modulus of the bias and panel (**B**) the corresponding phase uncertainty. For reference to the experimental parameters used in the main body of the article, we include a horizontal red dashed line indicating the experimental baseline fluctuations of $\sigma = 0.063$. As discussed in the article, the transition from a two-peak to a one-peak PDF occurs at $A \cong 1.78\sigma$. This threshold is indicated in Figs. S2 and S3 by the orange line and supports the claim that the ambiguity of the fit parameters A and σ leads to a bias and increased uncertainty in PEAC.

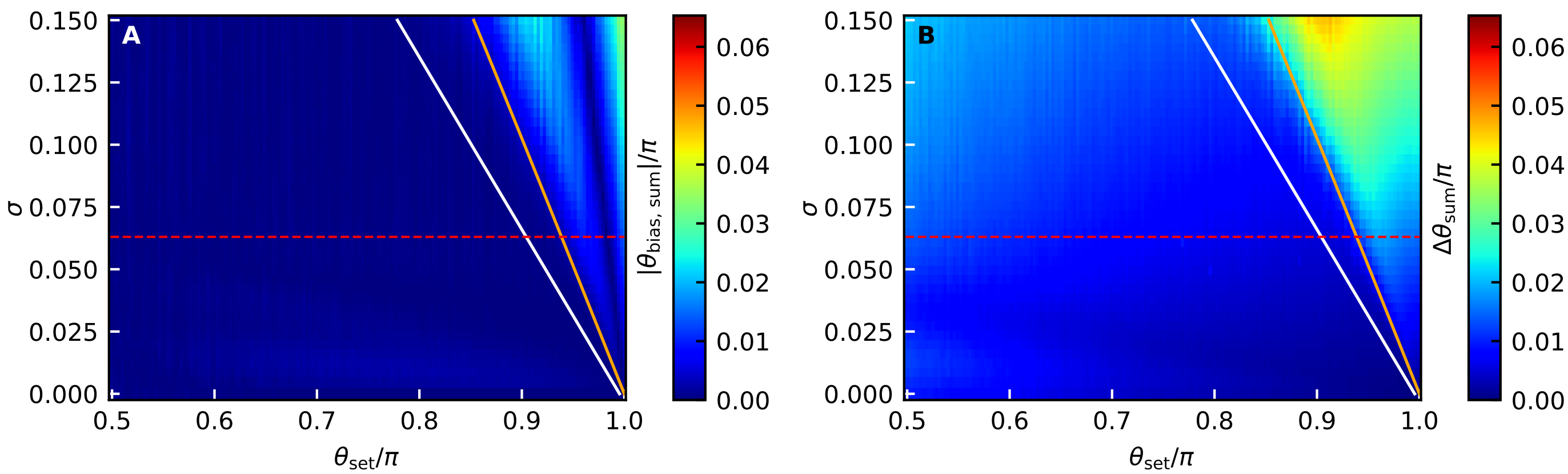

FIG. S2. **Baseline-noise scan for bias and uncertainty of PEAC.** The orange line indicates the criterion $A \cong 1.78\sigma$, while the white line highlights the working point of PEAC. The horizontal red dashed line at $\sigma = 0.063$ marks our experimental noise value. (**A**) Absolute value of the bias for PEAC along S_{sum}. (**B**) Phase uncertainty for PEAC along S_{sum}.

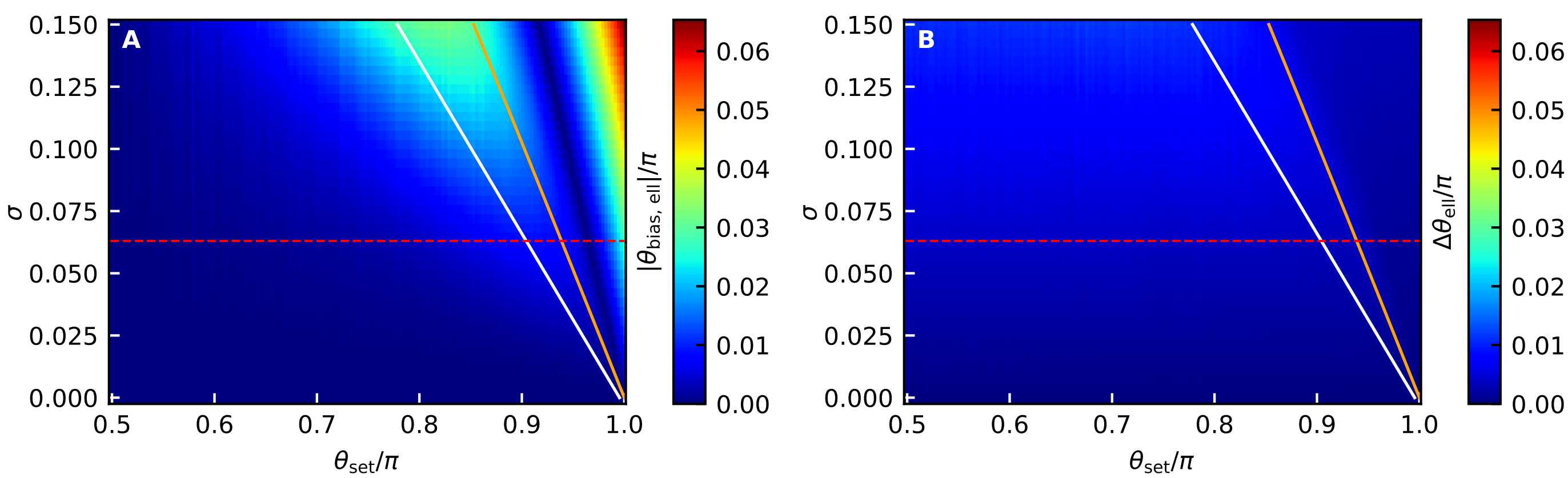

FIG. S3. **Baseline-noise scan for bias and uncertainty of ellipse fitting.** The orange line indicates the criterion $A \cong 1.78\sigma$, while the white line highlights the working point of PEAC. The horizontal red dashed line at $\sigma = 0.063$ marks our experimental noise value. (**A**) Absolute value of the bias for ellipse fitting. (**B**) Phase uncertainty for ellipse fitting.

In the given parameter region, we can linearise the $A \cong 1.78\sigma$ criterion, establishing a linear connection between the value of θ_{set} at which the transition occurs and σ, as confirmed by the orange line. The orange line essentially marks the region of substantial bias and uncertainty increase for PEAC along S_{sum}. In contrast, ellipse fits show a substantially larger bias over a broad range of θ_{set}, extending beyond the region bounded by the orange line. Nevertheless, the phase uncertainty of PEAC is larger than that of ellipse fitting, especially in the region around degeneracy where ellipses underestimate the systematic error induced by bias.

Additionally, we indicate with the white line the working point θ_{wp}, i. e. the phase of minimal phase uncertainty in PEAC. This working point always falls within the region of negligible bias, providing an uncertainty comparable to ellipse fits while maintaining reduced bias. By fitting a line to the minimum, we find that this behaviour can be approximated by $\theta_{\mathrm{wp}}/\pi \cong 1 - 1.45\sigma$ within our parameter regime. This trend is in agreement with the expectation that $\theta_{\mathrm{wp}} \to \pi$ for $\sigma \to 0$.

Moreover, we observe an advantage of PEAC at higher noise levels, in particular for our experimental value, while ellipses perform the best in low-noise environments. Thus, we used our numerical model to explore PEAC from high- to low-noise environments, highlighting its complementarity to ellipse fitting. These insights are supported by the criterion $A \cong 1.78\sigma$, which allows the identification of the effective bias-free region and the working point of PEAC.

S3. COMPLEMENTARITY OF PEAC AND ELLIPSE FITTING

If correlated data in the bivariate plane have been recorded, ellipse fits can be performed as described in the main body of the article. However, such data also allows PEAC to be applied in addition to, or in combination with, this analysis. PEAC is flexible in that, beyond the semi-axes along the diagonal and anti-diagonal, any projection angle α can be used for parameter extraction, as sketched in Fig. S4(**A**).

In fact, considering the general rotated signal $S(\alpha)$ with amplitude $A(\alpha) = A_0\sqrt{1+\sin(2\alpha)\cos\theta}$ (cf. Eq. (3) of the main text for $\lambda_0 = 0, \lambda_{-1} = \cos\alpha, \lambda_{+1} = \sin\alpha$), we can calculate its phase uncertainty for $\alpha \neq \pi/2\,\mathbb{Z}$

$$\Delta\theta(\alpha) = \frac{2\Delta A(\alpha)}{A_0}\frac{\sqrt{1+\sin(2\alpha)\cos\theta}}{|\sin(2\alpha)\sin\theta|}, \tag{S1}$$

where $\Delta A(\alpha)$ is the uncertainty of the rotated oscillation amplitude. Assuming that the variance ΔA is independent of α, the phase uncertainty for $\theta \in (\pi/2, 3\pi/2)$ becomes minimal for $\alpha = \pi/4$ implying $\lambda_{\pm 1} = 1/\sqrt{2}$, i. e. an equal superposition of the signals. In this sense S_{sum} is optimal since $\Delta\theta(\alpha) \geq \Delta\theta(\alpha = \pi/4)$. Under this assumption, the ellipse's principal axes S_{sum} and S_{diff} are the directions one should choose when employing PEAC for $\theta \in (\pi/2, 3\pi/2)$ and $\theta \in (3\pi/2, \pi)$, respectively.

Nevertheless, this analysis neglects the treatment of $\Delta A(\alpha)$, which in practice depends on the employed fitting routine as discussed in Sect. 2.3 of the main text. For this reason, we performed a numerical analysis by scanning the angle α as shown in Fig. S4(**A**), along which we generate histograms and perform PEAC. Here, we show in detail specific values of α allowing to assess PEAC's performance close to $\alpha = \pi/4$ and far away. In Fig. S4(**B**) and (**C**) we present bias and uncertainty, respectively. As expected from Eq. (S1), increasing deviations from $\alpha = \pi/4$ lead to larger bias and uncertainty. For values in the vicinity of $\pi/4$ we observe a behaviour similar to that along the semi axes. However, compared to $\alpha = \pi/4$, the onset of the bias occurs at smaller deviations from the degeneracy point and the uncertainty is even reduced in the vicinity of degeneracy. This effect can be understood as follows: By rotating around α, the two peaks of the PDF are projected on an axis where their separation is increased, such that the transition from a two-peaked to a single-peaked PDF occurs closer to degeneracy. Consequently, the fit routine can profit from a reduced ambiguity in A and σ when fitting the histogram's PDF. Exactly this benefit can be observed for α in vicinity, of but not at, $\pi/4$, as illustrated by the choice $\alpha = 0.249\pi$.

While other work on quantum clocks claims to gain orders of magnitude better uncertainty [51] when working close to, but not at $\alpha = \pi/4$, in our case, we do not observe a comparable improvement with PEAC. In addition, although the onset of the bias occurs closer to degeneracy, the magnitude of its variation is larger, as shown by the inset of Fig. S4(**B**). Crucially, θ_{bias} exceeds the statistical uncertainty $\Delta\theta$. As a consequence, $\theta_{\mathrm{rec}} \pm \Delta\theta$ does not include the true value and phase estimates cannot be trusted through the entire θ range. In contrast, an equal superposition with $\alpha = \pi/4$, i. e. S_{sum}, has a smaller bias close to degeneracy but an increased uncertainty so that the true value is always included within the uncertainty band of the estimated phase. The criterion $A < 1.78\sigma$, together with the freedom to choose α arbitrarily, allows optimising the analysis for a given experimental implementation. Thus, whenever correlated bivariate data are recorded, PEAC allows choosing α for an improved analysis tailored to the specific needs of the specific estimation task. These findings highlight the complementarity of PEAC to pure ellipse-fitting approaches.

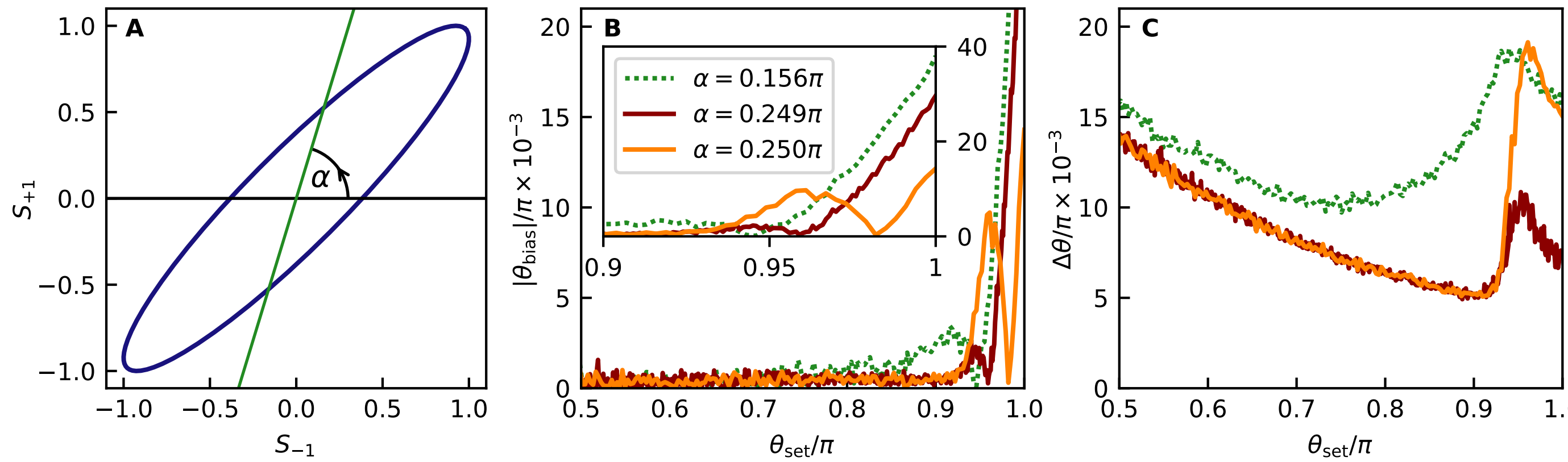

FIG. S4. **Rotated Projection Along the Ellipse.** (**A**) Schematic depiction of the projection through the data parametrised by the rotation angle α in the bivariate plane. Along this angle, the data is projected to obtain a histogram and to perform an analysis based on PEAC. Choosing $\alpha = \pi/4$ corresponds to the analysis performed with S_{sum}. (**B**) Resulting magnitude of the bias θ_{bias} for different choices of α. Increasing deviation from $\alpha = \pi/4$ leads to larger bias, whereas values in the vicinity of $\alpha = \pi/4$ show an extended bias-free region. Nevertheless, near degeneracy, $\alpha = \pi/4$ remains the favourable operating direction. (**C**) Uncertainty $\Delta\theta$ of reconstructed phase for different α. Increasing deviation from $\alpha = \pi/4$ induces a substantial increase of the phase uncertainty. In contrast, values close to $\alpha = \pi/4$ maintain the same minimal uncertainty at the working point, but exhibit reduced uncertainty closer to degeneracy compared to $\alpha = \pi/4$. Nevertheless, for $\alpha = 0.249\pi$, the bias exceeds the uncertainty, leaving $\alpha = \pi/4$ as the preferable choice near degeneracy.

A. Full Data Scan and Normalised Bias

Here, we present the complete data demonstrating PEAC's flexibility in choosing the rotation angle α. This freedom allows us to investigate the effect of population imbalances. We generate numerical datasets in which the parameters of the PDF match the experimental conditions $A_0 = 0.824$ and $\sigma = 0.063$. In contrast to the main article, we rely on histograms obtained by adding weighted signals $S(\alpha) = S_{-1}\cos\alpha + S_{+1}\sin\alpha$, which corresponds to histograms projected along the angle α. For each angle α, we perform a differential phase scan $\theta_{\mathrm{set}} \in [\pi/2, \pi]$ and use PEAC to extract the bias θ_{bias} and the phase uncertainty of the reconstructed phase $\Delta\theta$.

In the previous section, we have shown that $\alpha = \pi/4$ ($\alpha = 3\pi/4$) is optimal assuming that the uncertainty ΔA is independent of α, i. e. $\Delta\theta(\alpha) \geq \Delta\theta(\alpha = \pi/4)$. However, since $\Delta A(\alpha)$ may depend on the fit routine and the direction of projection, we explicitly investigate $\Delta\theta(\alpha)$ within our numerical model. We focus on the intervals $\theta_{\mathrm{set}} \in [\pi/2, \pi]$ and $\alpha \in [\pi/8, \pi/4]$ due to symmetries of S_{sum} and S_{diff} as well as the symmetry of exchanging S_{-1} and S_{+1}, respectively. The parameter scans are shown as density plots in Fig. S5, where panel (**A**) presents the magnitude of the bias and panel (**B**) the phase uncertainty. The black line marks the transition between a two-peak (left) and a single-peak (right) PDF; in the absence of such a line, the PDF is two-peaked throughout. Where applicable, the black line is determined by $A(\alpha, \theta) \cong 1.78\sigma$, corresponding to the generalised, α-dependent form of the criterion $A < 1.78\sigma$, with $A(\alpha, \theta) = A_0\sqrt{1 + \sin(2\alpha)\cos\theta}$. Thus, by tuning α, one can ensure a two-peaked PDF for any differential phase θ.

We observe an increase in both the bias and the uncertainty for values of α far from $\pi/4$. An increase of the uncertainty for deviations from $\pi/4$ is expected from Gaussian uncertainty propagation, as detailed above, which is supported by the trend shown in panel (**B**) from top to bottom. The uncertainty of $\Delta A(\alpha)$ becomes relevant in the vicinity of degeneracy. Near degeneracy, the bias shows large variations and can even change sign, producing a pronounced minimum at a narrow point, while the uncertainty remains substantial. Instead of focusing on this region, we identify the parameter regime of small bias. For $\alpha \to \pi/4$, the inset of panel (**A**) shows that the onset of the bias variation is closer to degeneracy for $\alpha \lesssim 0.249\pi$. Nevertheless, the bias at degeneracy is smaller for an equal superposition, i. e. $\alpha = \pi/4$.

Considering the uncertainty in panel (**B**), we find that its absolute minimum occurs at $\alpha = \pi/4$ shortly before $A(\alpha, \theta) = 1.78\sigma$, supporting the above claim. However, the inset shows that just before an equal superposition, the uncertainty is substantially reduced, so that already slight deviations from $\alpha = \pi/4$ lead to a reduced phase uncertainty. In connection to the section on the impact of baseline fluctuations, where we observed that the increase in uncertainty for $\alpha = \pi/4$ depends on σ, we expect this feature in turn also to depend on the magnitude of baseline fluctuations. In addition, for small population imbalances, the onset of bias variations shifts closer to degeneracy, accompanied by a reduced uncertainty. This finding suggests that operating with slight population imbalances, e. g. $\alpha = 0.249\pi$, may be advantageous. However, near degeneracy the bias variations also increase, which can introduce practical issues. To quantifies these effects, we introduce the normalised bias $|\theta_{\mathrm{bias}}|/\Delta\theta$.

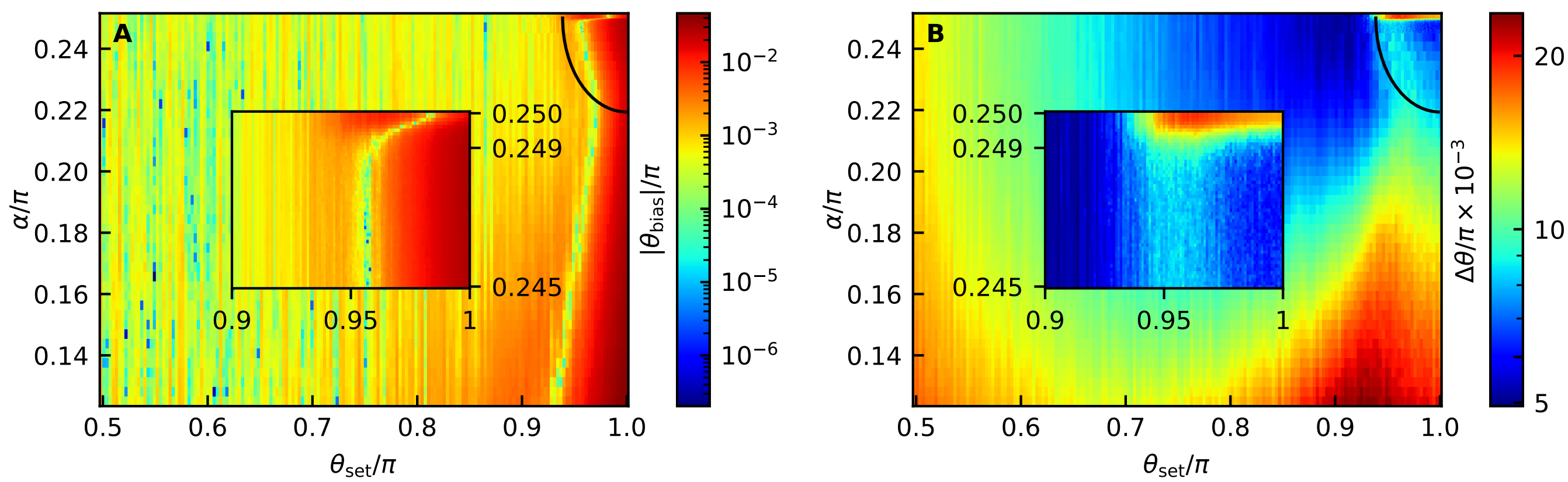

FIG. S5. **PEAC in the bivariate plane.** (**A**) Absolute value of the bias for PEAC in the α direction. (**B**) Phase uncertainty for PEAC in the α direction. In both panels, the black line marks the transition from a two-peak to a single-peak PDF. To the left of this line, the PDF exhibits two peaks, while to the right it shows a single peak.

The normalised bias is a measure for the quality of an estimate: If it exceeds unity, the statistical uncertainty underestimates the systematic uncertainty, and the one-standard-deviation interval $\theta_{rec} \pm \Delta\theta$ does not include the true value θ_{set}. Consequently, one ideally aims to operate in a regime where the normalised bias is less than or equal to unity. Figure S6(**A**) shows the normalised bias as a density plot for various angles α with respect to different θ_{set}. It becomes apparent that only for $\alpha = \pi/4$ the normalised bias remains less than unity (black line) over the whole range for θ_{set}; otherwise, it always exceeds unity when approaching degeneracy. In the inset of Fig. S6(**A**) we see the behaviour of the normalised bias in detail near degeneracy and equal superpositions.

For a detailed comparison, in Fig. S6(**B**) we also show cuts through the density plot at $\alpha = 0.250\pi$ and $\alpha = 0.249\pi$, together with the normalised bias obtained from geometric ellipse fitting. We find that the normalised bias for the geometric ellipse fit remains below unity up to 0.8π for our experimental parameters. In contrast, PEAC performs more favourably, both due to a reduced bias and an increased phase uncertainty. The normalised bias of PEAC for $\alpha = \pi/4$ is always smaller or close to unity throughout, such that an equal superposition does not underestimate the systematic error. Based on absolute values of bias and uncertainty, $\alpha = 0.249\pi$ might appear more beneficial, since the onset of bias variations shifts closer to degeneracy with a reduced uncertainty. However, at $\alpha = 0.249\pi$ the bias exceeds the uncertainty at degeneracy, so that the normalised bias surpasses unity. Thus, the optimal choice of α depends on the specific settings and fitting routine.

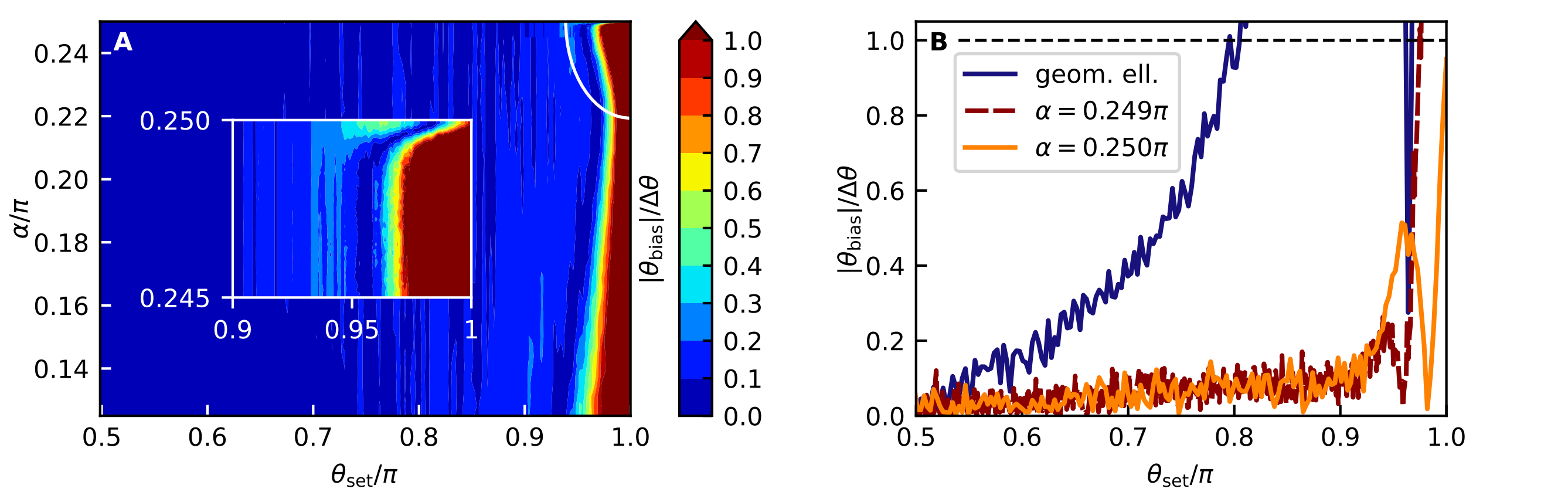

FIG. S6. **Normalised bias in the bivariate plane.** (**A**) Normalised bias for PEAC in the α direction. The inset shows a detailed plot of the upper right section where $0.245 \leq \alpha \leq 0.250$ and $9\pi/10 \leq \theta_{set} \leq \pi$. (**B**) Cuts through the density plot of the normalised bias for $\alpha = 0.249\pi$ (dark red, dashed), $\alpha = 0.250\pi$ (orange, solid), compared to the normalised bias for geometric ellipse fits (dark blue). In panel (**A**), the white line marks the transition from a two-peak to a single-peak PDF. To the left of the line, the PDF exhibits two peaks, while to the right it shows a single peak. In panel (**B**), the dashed black line at the top marks the threshold where $\theta_{bias}/\Delta\theta = 1$.

S4. MAXIMUM LIKELIHOOD ESTIMATION WITHIN PEAC

Different estimation techniques extracting the measurand from a given PDF or histogram are associated with different biases. For example, performing a least square (LSQ) fit of the PDF to a histogram, as in the main body of the article, is sensitive to the binning of the data, which effectively reduces the resolution and introduces an additional bias. Furthermore, the ambiguity of the fit parameters A and σ, especially for small values of A, e. g. close to the degeneracy point $\theta = \pi/2$, leads to an increase of bias and uncertainty.

Methods like maximum likelihood estimation (MLE) circumvent the binning of the data by directly evaluating the PDF at the measured data points through a specific likelihood function $\mathcal{L}(\hat{p}|\mathscr{s})$. In this Supplementary Materials, we implement an MLE approach using the negative log-likelihood function

$$\mathcal{L}(\hat{p}|\mathscr{s}) = -\sum_i \log\big(f_S(\hat{p}|\mathscr{s}_i)\big) \tag{S2}$$

and numerically minimise its value with respect to the parameter vector $\hat{p}$ using `scipy.optimize.minimize` and `scipy` package in `python` [100, 103].

This implementation has shown a substantial increase of computational cost with a runtime of 150 h compared to the LSQ fit approach (8 h) to fit our numerical data. In Fig. S7 we compare MLE and LSQ implementations of PEAC in bias (**A**) and uncertainty (**B**) for the summed signal S_{sum} of the numerical replication of the experiment. Additionally, we include bias and uncertainty for the geometric ellipse fits as a benchmark of standard evaluation techniques. Generally, we find that MLE indeed improves the performance of PEAC with a reduction both in bias and uncertainty, especially close to the degeneracy point $\theta = \pi$. The bias is reduced over the complete interval of θ, but the gain is small compared to the substantially increased computational cost. Around the degeneracy point, in-between the dashed lines, the bias is reduced by a factor of two, but exactly at degeneracy, the discontinuity is of the same order of magnitude as for the LSQ approach. Similar to the bias, the uncertainty is slightly reduced over the complete interval with the largest improvement close to the degeneracy. Outside the shown interval, the uncertainty is reduced by a factor of two at even multiples of π, where one would resort to using S_{diff} instead. In conclusion, we observe an improvement of the PEAC performance when using MLE compared to LSQ at the cost of substantially increased computational time in our implementation.

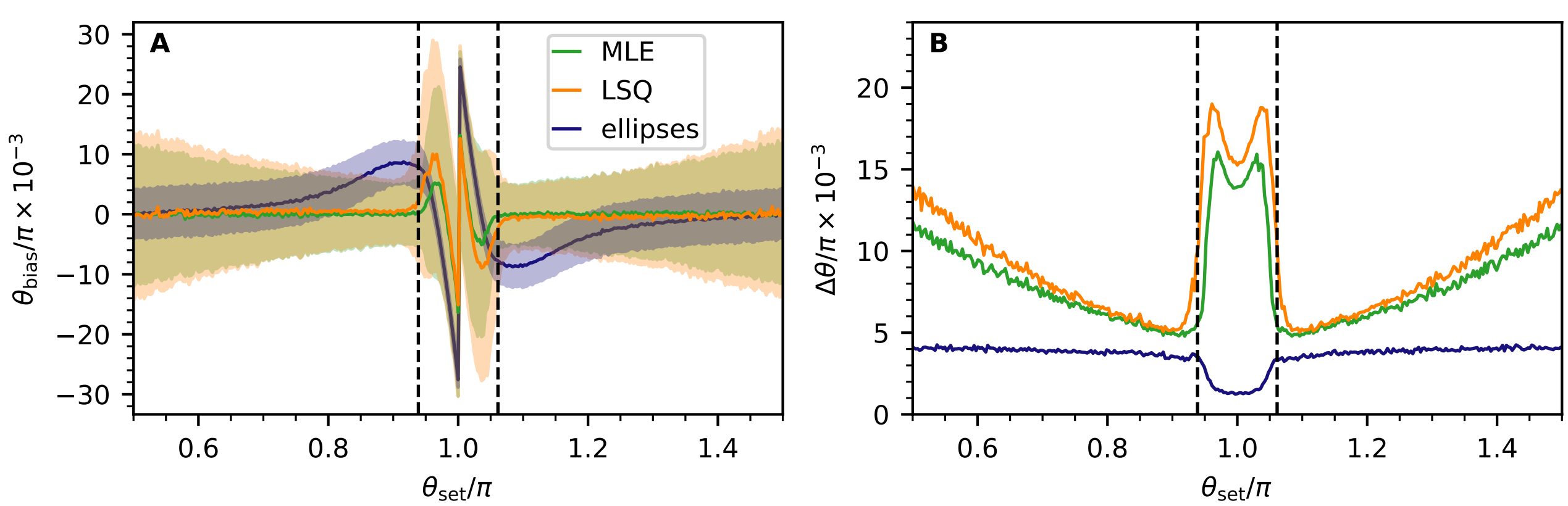

FIG. S7. **Comparison of least squares (LSQ) (orange), maximum likelihood (MLE) (green), and geometric ellipse fits (dark blue).** (**A**) Bias θ_{bias} for differential phases $\theta_{\mathrm{set}} \in [\pi/2, 3\pi/2]$ and three evaluation methods. As discussed in the main article, the LSQ approach of PEAC already substantially reduces bias compared to geometric ellipse fits at the cost of an increased uncertainty. Combining PEAC with the MLE approach bypasses the need for binning the data, i. e. calculating a histogram, and leads to a further reduction of the bias, while the qualitative trend is similar to the LSQ realisation. Inside the region marked by dashed lines, where $A < 1.78\sigma$, all three evaluation methods show an increase in bias, zero crossings, and apparent discontinuities. In the case of PEAC, such effects can be partially explained by the ambiguity of the fitting parameters A and σ, see the discussion in the main article. In contrast, the geometric ellipse fits converge at these points to the maximum value of the semi axes, i. e. $\sqrt{2}$. (**B**) Uncertainty $\Delta\theta$ for the three evaluation methods. Similar to the bias, we observe a slight reduction of the uncertainty for the MLE compared to the LSQ approach of PEAC. As expected, the geometric ellipse fits show a smaller uncertainty, being the more precise evaluation method albeit a larger bias and thus a reduced accuracy. Inside the dashed lines, $\Delta\theta$ is even reduced for the ellipse fits, as the fit parameters converge to the upper constraint.

S5. ACCURACY ASSESSMENT IN BIAS-UNCERTAINTY SPACE

In Fig. S7, we present both bias and uncertainty as function of the set phase θ_{set}, which can be identified as systematic and statistical errors. In experimental data analysis, the objective is generally to provide the smallest overall error budget by balancing both systematic and statistical contributions. However, there is no unique metric for combining these two quantities into a single performance measure: For example, equally weighing both contributions leads to the mean squared error $\theta_{\text{bias}}^2 + \Delta\theta^2$, but this metric does not distinguish between situations in which the bias exceeds the statistical uncertainty, so that the true value lies outside the error margin and the uncertainty band shown in Fig. S7 (A).

To enable a fair comparison of PEAC-MLE, PEAC-LSQ, and ellipse fitting, we avoid combining trueness, quantified by the bias, and precision, quantified by the standard deviation, into a single performance metric. Instead, Fig. S8 presents the overall error budget by plotting the uncertainty of each data point as a function of its bias for $\theta_{\text{set}} \in [\pi/2, 3\pi/2]$. The cone enclosed by the solid lines contains all measurements for which the set value lies within the reported 1σ uncertainty interval of the estimated mean. Estimates inside this cone therefore satisfy one criterion for true parameter estimation. While 100 % of the PEAC-LSQ results (and 99.7 % of the PEAC-MLE results) lie within this cone, only 62 % of the ellipse-fit results satisfy this criterion. The ellipse-fit points inside the cone correspond primarily to set phases located around the circular configuration, whereas the lower branch of ellipse-fit data originates from operating points close to the degeneracy.

The scatter plot also highlights the different characteristics of the estimators: PEAC substantially suppresses the bias and confines the results to a compact region of parameter space, whereas ellipse fits exhibit widespread bias, albeit with a generally smaller statistical uncertainty. Finally, the uncertainties of PEAC approach the uncertainty baseline achieved by the ellipse fits. This demonstrates that, when operating close to the working point, PEAC is competitive with ellipse fitting in all aspects, while consistently providing true estimates over all parameters and set phases of the interval.

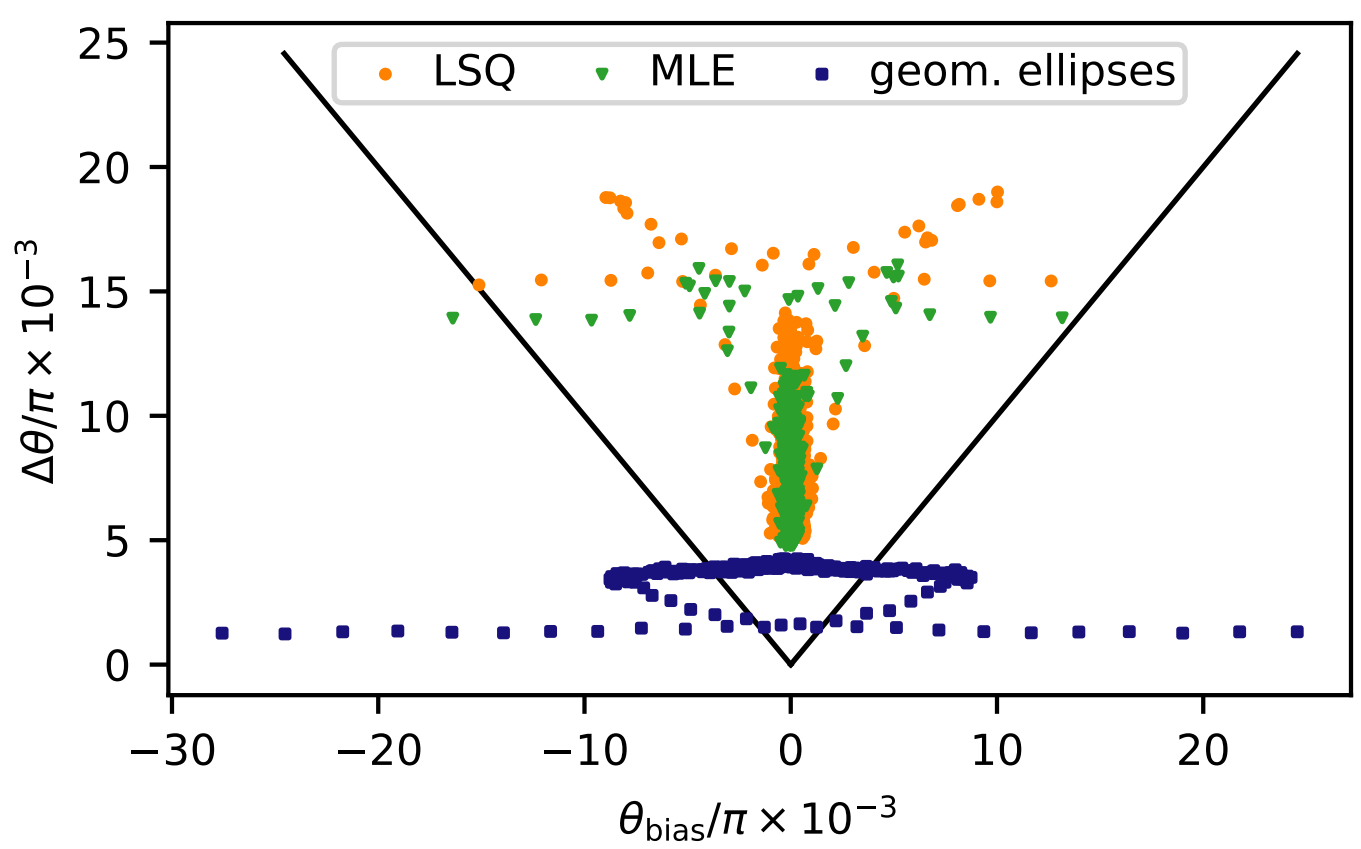

FIG. S8. **Comparison of differential phase estimators in the θ_{bias} and $\Delta\theta$ plane.** Scatter plot of bias versus uncertainty for each set phase $\theta_{\text{set}} \in [\pi/2, 3\pi/2]$ obtained from ellipse fitting (blue squares), PEAC-LSQ (yellow circles), and PEAC-MLE (green triangles). The solid lines denote the boundary $|\theta_{\text{bias}}| = \Delta\theta$. Points inside the cone correspond to estimates whose reported uncertainty interval contains the set value and can be regarded as true estimates. While 100 % of the PEAC-LSQ results (and 99.7 % of the PEAC-MLE results) lie within this cone, only 62 % of the ellipse-fit results satisfy this criterion.
